# Conditional recurrence-free survival after curative esophagectomy: individual patient data analysis of 11 trials

**DOI:** 10.1093/jnci/djaf347

**Published:** 2026-01-08

**Authors:** Jun Okui, Satoru Matsuda, Kengo Nagashima, Yasunori Sato, Hirofumi Kawakubo, Thomas Ruhstaller, Peter Thuss-Patience, Magnus Nilsson, Fredrik Klevebro, Lijie Tan, Shaoyuan Zhang, Thomas Aparicio, Guillaume Piessen, Charlène van der Zijden, Bianca Mostert, Bas P L Wijnhoven, Takahiro Tsushima, Hiroya Takeuchi, Ken Kato, Yuko Kitagawa

**Affiliations:** Department of Surgery, Keio University School of Medicine, Tokyo, Japan; Department of Biostatistics, Keio University School of Medicine, Tokyo, Japan; Department of Surgery, Keio University School of Medicine, Tokyo, Japan; Biostatistics Unit, Clinical and Translational Research Center, Keio University Hospital, Tokyo, Japan; Department of Biostatistics, Keio University School of Medicine, Tokyo, Japan; Department of Surgery, Keio University School of Medicine, Tokyo, Japan; Competence Center, Swiss Cancer Institute, Bern, Switzerland; Faculty of Medicine, University of Basel, Basel, Switzerland; Department of Hematology, Oncology, and Cancer Immunology, Charité-University Medicine Berlin, Campus Virchow Klinikum, Berlin, Germany; Department of Upper Abdominal Diseases, Karolinska University Hospital, Stockholm, Sweden; Division of Surgery and Oncology, Department of Clinical Science, Intervention and Technology (CLINTEC), Karolinska Institutet, Stockholm, Sweden; Department of Upper Abdominal Diseases, Karolinska University Hospital, Stockholm, Sweden; Division of Surgery and Oncology, Department of Clinical Science, Intervention and Technology (CLINTEC), Karolinska Institutet, Stockholm, Sweden; Department of Thoracic Surgery, Zhongshan Hospital, Fudan University, Shanghai, China; Department of Thoracic Surgery, Zhongshan Hospital, Fudan University, Shanghai, China; Department of Digestive Oncology, Hôpital Saint-Louis, APHP, Université Paris Cité, Paris, France; UMR9020-U1277, CANTHER—Cancer Heterogeneity, Plasticity and Resistance to Therapies, University Lille, CNRS, Inserm, CHU Lille, Lille, France; Department of Surgery, Erasmus MC Cancer Institute, Erasmus University Medical Center, Rotterdam, The Netherlands; Department of Medical Oncology, Erasmus MC Cancer Institute, Erasmus University Medical Center, Rotterdam, The Netherlands; Department of Surgery, Erasmus MC Cancer Institute, Erasmus University Medical Center, Rotterdam, The Netherlands; Division of Gastrointestinal Oncology, Shizuoka Cancer Center, Shizuoka, Japan; Department of Surgery, Hamamatsu University School of Medicine, Shizuoka, Japan; Department of Head and Neck, Esophageal Medical Oncology, National Cancer Center Hospital, Tokyo, Japan; Department of Surgery, Keio University School of Medicine, Tokyo, Japan

## Abstract

**Background:**

Most recurrences after curative surgery for esophageal cancer occur within 2 years. Conventional recurrence-free survival (RFS), calculated from the time of surgery, may underestimate prognosis for patients who remain recurrence-free during the early postoperative years. This study aimed to evaluate conditional RFS and recurrence timing to inform individualized follow-up strategies.

**Methods:**

An individual patient data (IPD) analysis was conducted using randomized controlled trials (RCTs) comparing perioperative treatments for resectable esophageal or gastroesophageal junction cancer. Conditional RFS, defined as the probability of remaining recurrence-free for an additional *y* years given *x* years already survived without recurrence (RFS_*y*_|RFS_*x*_), was estimated.

**Results:**

IPD from 10 phase III and 1 phase II RCTs were analyzed (*n* = 2268 patients with R0 resection). In squamous cell carcinoma (SCC), RFS_5_|RFS_0_ was 47.9%, which increased to 63.0%, 72.5%, 78.2%, and 81.2% at RFS_5_|RFS_1–4_. Among patients who recurred, 58.8% of pN-positive cases recurred within 1 year and 81.7% within 2 years, compared with 42.8% and 69.9% in pN0. At baseline (RFS_5_|RFS_0_), patients with pN-positive disease or pM1 disease had worse 5-year RFS than those with pN0 or pM0 disease. However, among patients who remained recurrence-free for 4 years after surgery (RFS_5_|RFS_4_), the pattern was reversed, with advanced groups showing better subsequent 5-year RFS. Similar trends were observed in adenocarcinoma.

**Conclusions:**

Conditional RFS improves over time, particularly in advanced-stage esophageal cancer. Although advanced cases are typically monitored more intensively, findings suggest comparable follow-up intensity may be appropriate once patients remain recurrence-free for a certain postoperative period.

## Introduction

Esophageal cancer ranks 11th in global cancer incidence and seventh in mortality.^1^ Because esophageal cancer can spread from the neck to the abdomen even during early stages, leading to systemic progression,^2^^,^^3^ multidisciplinary treatment including surgery, chemotherapy, radiotherapy, and immunotherapy is required.^4-6^ Despite this multidisciplinary treatment, the risk of recurrence remains high; several studies have reported that approximately 40%-60% of patients experience recurrence after curative resection for esophageal cancer.^7-9^ However, no definitive evidence supports the utility or optimal strategy for routine follow-up, and standardized surveillance protocols have not yet been established.^10^

Many studies demonstrated that 80%-90% of recurrences after curative esophagectomy occur within the first 2 years postoperatively.^8^^,^^11^ Similarly, in many other malignancies, the hazard rates for death or recurrence remain relatively high during the initial years after diagnosis but then decrease markedly.^12^ This pattern suggest that patients who remain recurrence-free during the early postoperative period may have a more favorable long-term prognosis. However, conventional recurrence-free survival (RFS), calculated from the time of surgery, may underestimate the prognosis for such patients. To address this, conditional RFS—defined as the probability of remaining recurrence-free for an additional *y* years, given that the patient has already remained recurrence-free for *x* years (RFS_*y*_|RFS_*x*_)—provides a more appropriate analytic approach. Conditional RFS, grounded in the concept of conditional probability, better reflects how changing hazard rates over time influence prognosis. In this way, conditional survival (CS) analysis offers more meaningful prognostic information by providing dynamic estimates of future survival probabilities for patients who have already survived initial cancer treatment.

Previous studies in melanoma and colon cancer have shown that conditional RFS improves over time, with the trend being more pronounced in patients with advanced pathological tumor-node-metastasis (pTNM) stages compared with those who have less advanced disease.^12^^,^^13^ This observation indicates that in patients with advanced cancer, recurrence—when it occurs—tends to arise relatively early. Such findings hold essential importance for optimizing follow-up intervals. However, to date, no study has evaluated conditional RFS in patients with esophageal cancer, particularly using individual patient data (IPD) from randomized controlled trials (RCTs). Although patients with advanced-stage esophageal cancer typically undergo more intensive surveillance after surgery, a clearer understanding of recurrence dynamics may help prevent unnecessarily prolonged or uniform follow-up strategies.

Therefore, this study aimed to evaluate conditional RFS and recurrence timing to explore the potential for individualizing follow-up intervals in patients with surgically resectable advanced esophageal cancer. Furthermore, the study sought to provide insights to guide survivorship care for patients who, despite being initially diagnosed with advanced-stage disease, remained recurrence-free over the long term.

## Methods

### Study design

This study conducted an integrated analysis of IPD from RCTs that compared perioperative therapies for surgically resectable advanced esophageal and gastroesophageal junction cancer (International Collaborative Research Project, “JP-ESOP23”).^14^^,^^15^ This study was approved by the Keio University School of Medicine Ethics Committee (approval number: 2023-1032). The study was performed in accordance with the Declaration of Helsinki. The requirement for informed consent was waived due to the retrospective nature of the study. This study adhered to the Strengthening the Reporting of Observational Studies in Epidemiology (STROBE) guidelines.^16^

A systematic review was initially conducted, and requests for IPD were sent to the principal investigators of all eligible trials. The review was registered in PROSPERO (registration number: CRD42023396321), with further details provided elsewhere.^14^ Trials were considered eligible if they were phase III RCTs evaluating perioperative therapies for resectable esophageal or gastroesophageal junction cancer and had closed to patient accrual before December 31, 2020. Trials that compared perioperative therapies with immune checkpoint inhibitors were excluded to enhance internal validity, because only a few phase III trials for resectable esophageal cancer were available or had published results at the time of planning.

### Patient selection and data collection

The inclusion criteria for IPD were as follows: (1) older than 18 years; (2) diagnosis of thoracic esophageal cancer or esophagogastric junction cancer; (3) histological confirmation of adenocarcinoma, squamous cell carcinoma (SCC), adenosquamous carcinoma, or basaloid cell carcinoma; (4) clinical stage I, II, or III disease (excluding cT1N0 and cT4b) according to the 8th edition of the UICC-TNM classification^17^; and (5) achievement of R0 resection. Patients were excluded if they opted out of the study or if data on overall survival (OS) or RFS were unavailable.

Data on patient characteristics, clinicopathological factors, and surgical procedures were collected from the IPD of each trial. Pathological response was defined as either pathological grade (pGrade)^18^^,^^19^ or Mandard tumor regression grade (TRG),^20^ depending on the trial. Pathological complete response (pCR) was defined as ypT0N0. Overall survival was calculated from the date of randomization to the date of death or last follow-up. RFS was calculated from the date of randomization to the date of death, recurrence, or last follow-up. All data were reanalyzed centrally and checked for inconsistencies. In this study, because the cohort included both patients who received preoperative treatment and those who did not, the pathological stage (pStage) was consistently denoted as (y)pStage.

Recent clinical trials on resectable esophageal cancer have focused primarily on adjuvant therapy in patients with R0 resection.^21^ Therefore, this analysis targeted that population and emphasized RFS.^22^ Moreover, because this study represented a secondary evaluation of completed RCTs, unified criteria for assessing progression-free survival or disease-free survival across studies could not be established. To preserve internal validity, RFS after R0 resection was selected as the endpoint. In addition, because randomization may have been conducted postoperatively in certain trials, the date of randomization, rather than that of surgery, was uniformly used as the reference time point (*t_0_*) across all studies.

### Definition of conditional survival

The survival function, denoted as S(*t*), represents the probability that a patient remains alive *t* years from a reference point (eg, the time of diagnosis). However, this function does not capture changes in prognosis over time and is considered a “static” prediction. Patients who have already survived for several years—and their physicians—are often more interested in the dynamic probability of surviving an additional *t* years, given survival up to a certain point *s* years. This dynamic probability is referred to as CS. Let S(*t*) denote the probability of surviving up to time *t* and *h(t)* the instantaneous hazard rate at time *t*. Then, CS(*y*|*x*), the probability of surviving an additional *y* years given survival for *x* years, is defined as


$$CS(y|x)=\frac{S(x+y)}{S(x)}=\exp\left(-\int_{x}^{x+y}h(t)\;dt\right)$$

Notably, when *x *= 0, CS(*t* | 0) is equivalent to the survival function S(*t*), because S(0) = 1. In principle, CS can be calculated using the standard Kaplan-Meier estimate $\hat{S}(t)$ as follows:


$$\hat{CS}(t|s)=\frac{\hat{S}(s+t)}{\hat{S}(s)}$$

For example, CS(5|2) can be estimated by dividing the Kaplan-Meier estimate at *t *= 7 by the estimate at *t *= 2. An equivalent approach involves restricting the analysis to patients who remain alive and uncensored at *s *= 0, 1, 2, 3, or 4 years, and then performing Kaplan-Meier estimation with each time point treated as a new origin. This method, known as the conditional Kaplan-Meier approach, was adopted in this study because both approaches yield identical estimates of CS.^12^^,^^23^

### Statistical analysis

Conditional 5-year recurrence-free survival (RFS_5_|RFS_*x*_) was estimated at annual landmark time points ranging from 0 to 4 years after randomization. Conditional RFS was defined as the probability of remaining recurrence-free for an additional 5 years, given that a patient had remained recurrence-free up to a specified landmark. The maximum landmark was set at 4 years because this study represents a secondary analysis of completed RCTs, most of which had follow-up periods shorter than 10 years. Moreover, to identify the peak timing of recurrence, cumulative incidence distributions were estimated. This analysis was restricted to patients in the overall cohort who experienced recurrence during follow-up, and the cumulative incidence was plotted over time. Censored patients were excluded from this analysis.

The Aalen additive hazards model was applied to evaluate time-dependent changes in the effects of clinical variables on the hazard of RFS.^24^^,^^25^ In contrast to the Cox proportional hazards model, the Aalen model allows covariates to influence the hazard additively and in a time-dependent manner, providing enhanced flexibility when handling nonproportional hazards. The model was estimated using the *aalen* function in the R package “timereg.”^26^ To visualize how covariate effects evolved during the postoperative course, cumulative time-dependent coefficients with 95% confidence intervals (CIs) were plotted. To depict changes in hazard ratios with (y)pTNM as covariates, all considered variables were converted into binary variables. This approach was particularly aided in identifying whether the prognostic impact of each factor varied over time.

Subgroup analyses were performed for all analyses described above, stratified by histological subtype (adenocarcinoma or SCC) and key clinicopathological factors, including pathological T/N/M stage, pCR, TRG,^20^ and pGrade.^18^^,^^19^ All analyses were conducted using R software version 4.5.1 (R Foundation for Statistical Computing, Vienna, Austria). Missing values were not imputed.

## Results

The literature search, study selection, and IPD selection process are shown in Figure S1. Of the 22 eligible trials, 10 provided IPD.^27-36^ For the CMISG1701 trial,^28^ IPD were available only for patients enrolled at certain facilities. Additionally, the present analysis included 1 large phase II trial in which participating institutions provided supplementary IPD.^37^ Although this trial was not originally designed to compare the efficacy or safety of interventions, inclusion was considered appropriate because its primary objective was to conduct general survival analyses in patients with esophageal cancer (Table S1). A total of 2268 patients with clinical stages I, II, and III (excluding cT1N0 and cT4b) who underwent R0 resection were included (Figure S1). The patient characteristics are summarized in Table 1. Histologically, 1597 patients had SCC, whereas 664 had adenocarcinoma. Details regarding the numbers of deaths, recurrence events, and the distribution of recurrence patterns are provided in Table S2, the median follow-up duration for each conditional cohort is summarized in Table S3, and the data of cancer-related and non-cancer-related deaths over time are presented in Table S4.

**Table 1.** Patient characteristics.

	Overall	SCC	Adenocarcinoma
**N**	2268	1597	664
**Trial**			
** Boonstra et al.**	78 (3.4%)	78 (4.9%)	0 (0%)
** CMISG1701**	70 (3.1%)	70 (4.4%)	0 (0%)
** CROSS**	259 (11.4%)	62 (3.9%)	191 (28.8%)
** FFCD9102**	98 (4.3%)	89 (5.6%)	9 (1.4%)
** FFCD9901**	125 (5.5%)	88 (5.5%)	36 (5.4%)
** JCOG1109**	532 (23.5%)	532 (33.3%)	0 (0%)
** JCOG9204**	220 (9.7%)	220 (13.8%)	0 (0%)
** JCOG9907**	297 (13.1%)	297 (18.6%)	0 (0%)
** NeoRes**	123 (5.4%)	34 (2.1%)	89 (13.4%)
** NeoRes2**	218 (9.6%)	44 (2.8%)	174 (26.2%)
** SAKK75/08**	248 (10.9%)	83 (5.2%)	165 (24.8%)
**Age, mean (SD)**	61.4 (8.1)	61.3 (7.9)	61.7 (8.5)
**Sex**			
** Female**	472 (20.8%)	261 (16.3%)	211 (31.8%)
** Male**	1796 (79.2%)	1336 (83.7%)	453 (68.2%)
**PS**			
** 0**	1580 (76.9%)	1068 (75.9%)	506 (79.2%)
** 1**	474 (23.1%)	340 (24.1%)	133 (20.8%)
**Tumor location**			
** EC**	1784 (87.1%)	1358 (98.6%)	420 (63.3%)
** GEJC**	264 (12.9%)	19 (1.4%)	244 (36.7%)
**Ct**			
** 1**	74 (4.0%)	72 (6.0%)	2 (0.3%)
** 2**	371 (20.1%)	236 (19.5%)	135 (21.5%)
** 3**	1350 (73.3%)	875 (72.4%)	470 (74.8%)
** 4a**	47 (2.6%)	25 (2.1%)	21 (3.3%)
**cN**			
** 0**	697 (31.9%)	445 (29.4%)	250 (37.8%)
** 1**	1102 (50.5%)	813 (53.7%)	284 (43.0%)
** 2**	223 (10.2%)	173 (11.4%)	50 (7.6%)
** 3**	31 (1.4%)	16 (1.1%)	15 (2.3%)
** cN+**	130 (6.0%)	68 (4.5%)	62 (9.4%)
**cStage**			
** I**	50 (2.6%)	50 (3.9%)	0 (0%)
** II**	685 (35.8%)	536 (42.1%)	148 (23.1%)
** III**	1180 (61.6%)	688 (54.0%)	492 (76.9%)
**Perioperative treatment**			
** NAC**	643 (28.4%)	601 (37.6%)	42 (6.3%)
** NACRT**	1048 (46.2%)	523 (32.7%)	521 (78.5%)
** Surgery alone**	320 (14.1%)	216 (13.5%)	101 (15.2%)
** Adjuvant chemotherapy**	257 (11.3%)	257 (16.1%)	0 (0%)
**(y)pT**			
** 0**	436 (20.8%)	292 (19.4%)	143 (24.6%)
** is**	15 (0.7%)	11 (0.7%)	4 (0.7%)
** 1**	364 (17.4%)	278 (18.5%)	86 (14.8%)
** 2**	330 (15.8%)	216 (14.3%)	112 (19.3%)
** 3**	919 (43.9%)	685 (45.5%)	230 (39.6%)
** 4**	30 (1.4%)	24 (1.6%)	6 (1.0%)
**(y)pN**			
** 0**	1127 (49.9%)	737 (46.4%)	385 (58.2%)
** 1**	815 (36.1%)	652 (41.0%)	161 (24.3%)
** 2**	142 (6.3%)	109 (6.9%)	33 (5.0%)
** 3**	63 (2.8%)	38 (2.4%)	25 (3.8%)
** pN+**	112 (5.0%)	54 (3.4%)	58 (8.8%)
**(y)pM1**	122 (5.4%)	122 (7.7%)	0 (0%)
**(y)pStage**			
** I**	732 (35.2%)	474 (31.7%)	256 (44.3%)
** II**	312 (15.0%)	216 (14.5%)	93 (16.1%)
** III**	893 (43.0%)	676 (45.3%)	215 (37.2%)
** IV**	141 (6.8%)	127 (8.5%)	14 (2.4%)
**pCR (ypT0N0)**			
** pCR**	369 (22.0%)	244 (21.8%)	124 (22.3%)
** non-pCR**	1309 (78.0%)	873 (78.2%)	433 (77.7%)
**pGrade**			
** 0**	47 (7.0%)	47 (7.0%)	NA
** 1a**	236 (35.3%)	236 (35.3%)	NA
** 1 b**	85 (12.7%)	85 (12.7%)	NA
** 2**	171 (25.6%)	171 (25.6%)	NA
** 3**	130 (19.4%)	130 (19.4%)	NA
**TRG**			
** 1**	266 (31.3%)	129 (43.9%)	136 (24.6%)
** 2**	235 (27.6%)	62 (21.1%)	173 (31.3%)
** 3**	148 (17.4%)	33 (11.2%)	114 (20.7%)
** 4**	201 (23.6%)	70 (23.8%)	129 (23.4%)

Values are *n* (%) unless otherwise indicated.

Abbreviations: EC = esophageal cancer; GEJC = gastroesophageal junction cancer; NAC = neoadjuvant chemotherapy; NACRT = neoadjuvant chemoradiotherapy; pCR = pathological complete response; pGrade = pathological grade; PS = performance status; SCC = squamous cell carcinoma; TRG = tumor regression grade.

**Figure 1.**
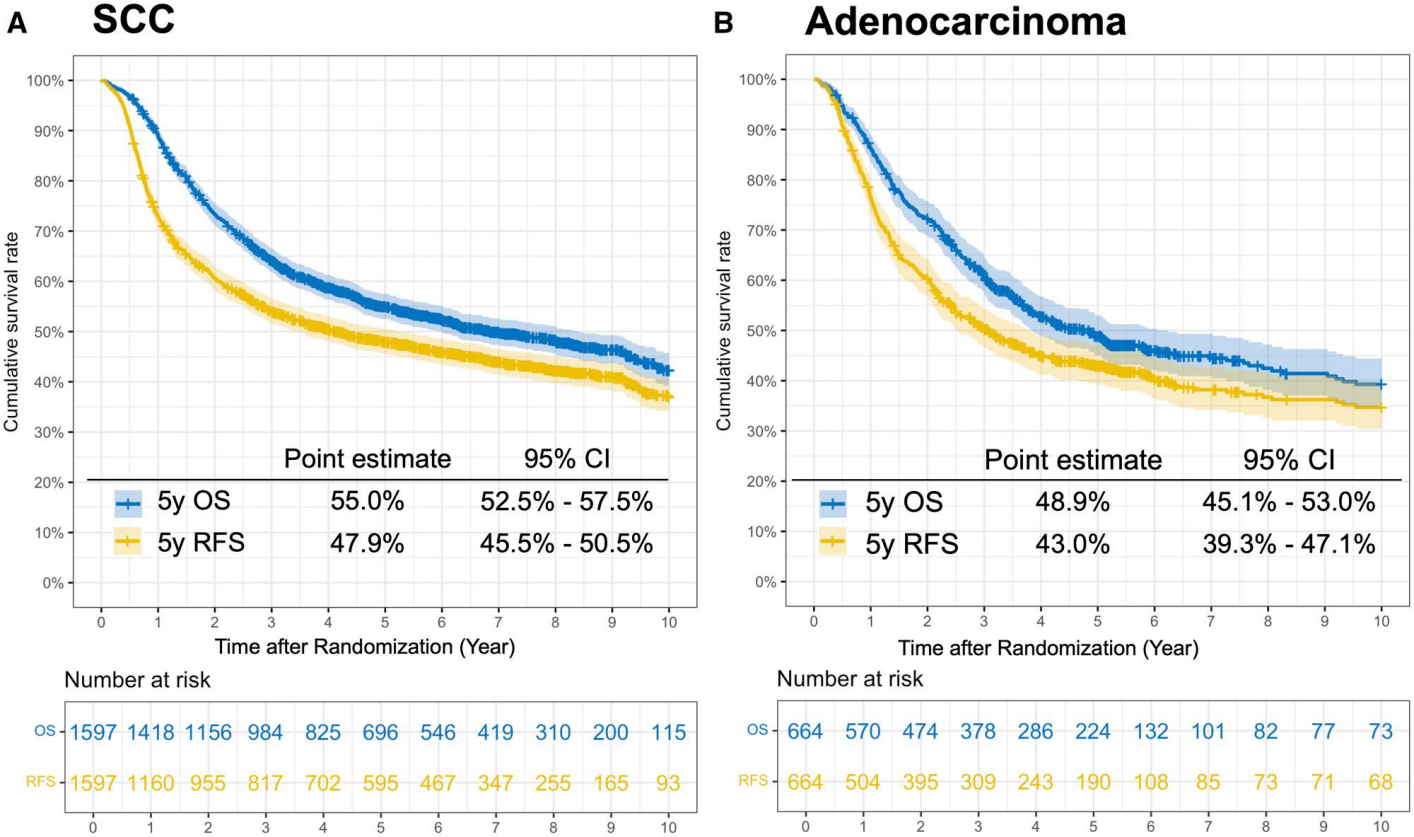
Kaplan-Meier estimates of overall and recurrence-free survival Shaded areas represent 95% confidence intervals (CIs). Abbreviations: OS = overall survival; RFS = recurrence-free survival; SCC = squamous cell carcinoma.

**Figure 2.**
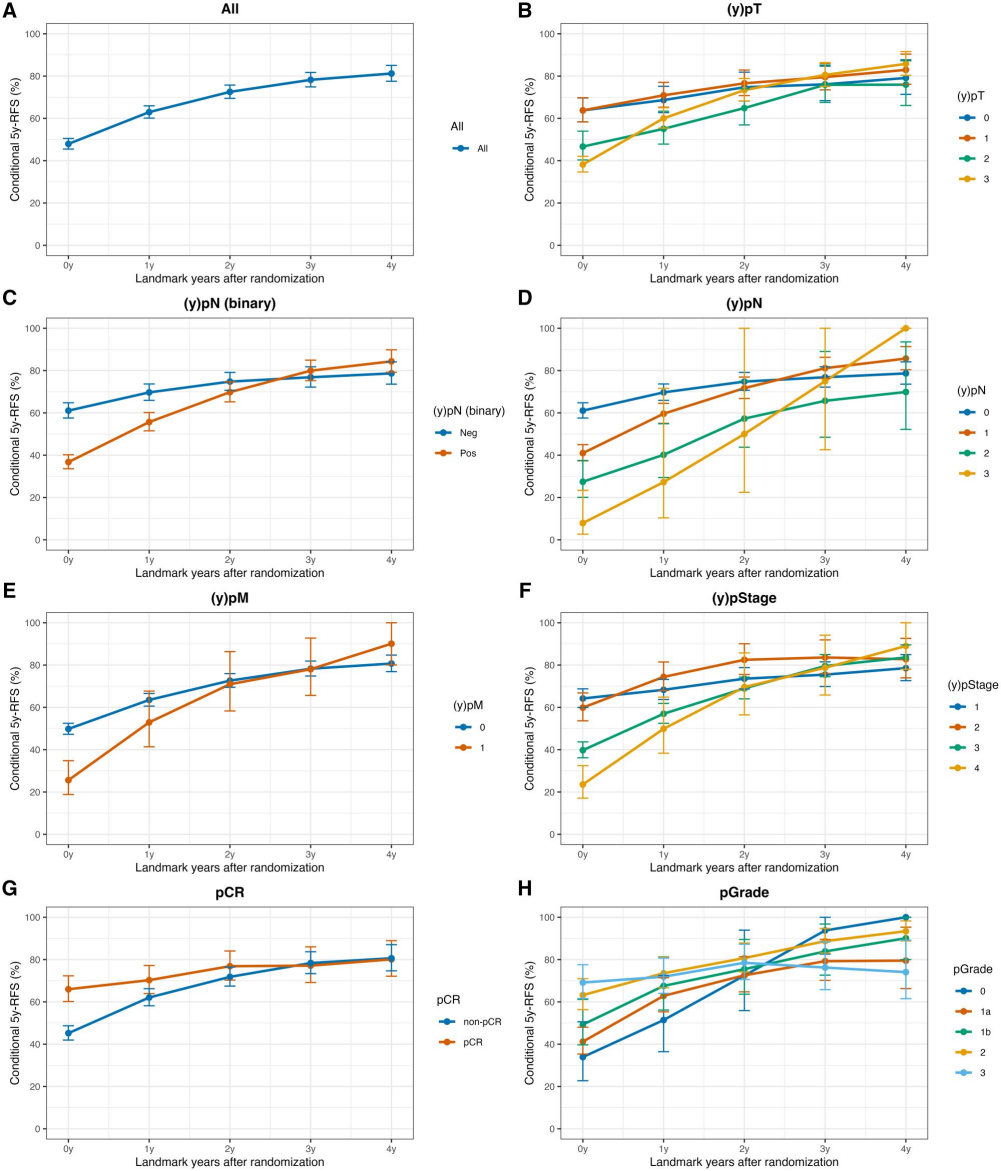
Conditional 5-year recurrence-free survival by landmark time and subgroup in patients with squamous cell carcinoma. Abbreviations: pCR = pathological complete response; pGrade = pathological grade; RFS = recurrence-free survival; TRG = tumor regression grade.

Patients with SCC had median OS and RFS of 6.8 years (95% CI = 6.0 to 8.4) and 4.1 years (95% CI = 3.4 to 5.3), respectively. In contrast, patients with adenocarcinoma had median OS and RFS of 4.8 years (95% CI = 3.9 to 6.2) and 3.1 years (95% CI = 2.5 to 3.8), respectively (Figure 1). The 5-year RFS rate (RFS_5_|RFS_0_) for patients with SCC was 47.9%; however, RFS_5_|RFS_1_, RFS_2_, RFS_3_, and RFS_4_ gradually increased to 63.0%, 72.5%, 78.2%, and 81.2%, respectively (Figure 2A). When comparing (y)pN-positive category with (y)pN0 and patients with (y)pM1 vs (y)pM0 category, RFS_5_|RFS_0_ was markedly worse in the former groups. However, by the time patients reached RFS_5_|RFS_4_, survival probabilities converged, and the relationship reversed (Figure 2C-E). Similar patterns were observed for (y)pT and (y)pStage (Figure 2B and F). Results for patients with adenocarcinoma are shown in Figure S2, demonstrating the same pattern. The subgroup analyses by pCR, TRG, and pGrade showed that although the comparison between patients with pCR and those without pCR demonstrated trends similar to those observed in advanced disease, TRG and pGrade exhibited consistent patterns with only minor fluctuations (Figure 2G-H and Figure S2G-H).

**Figure 3.**
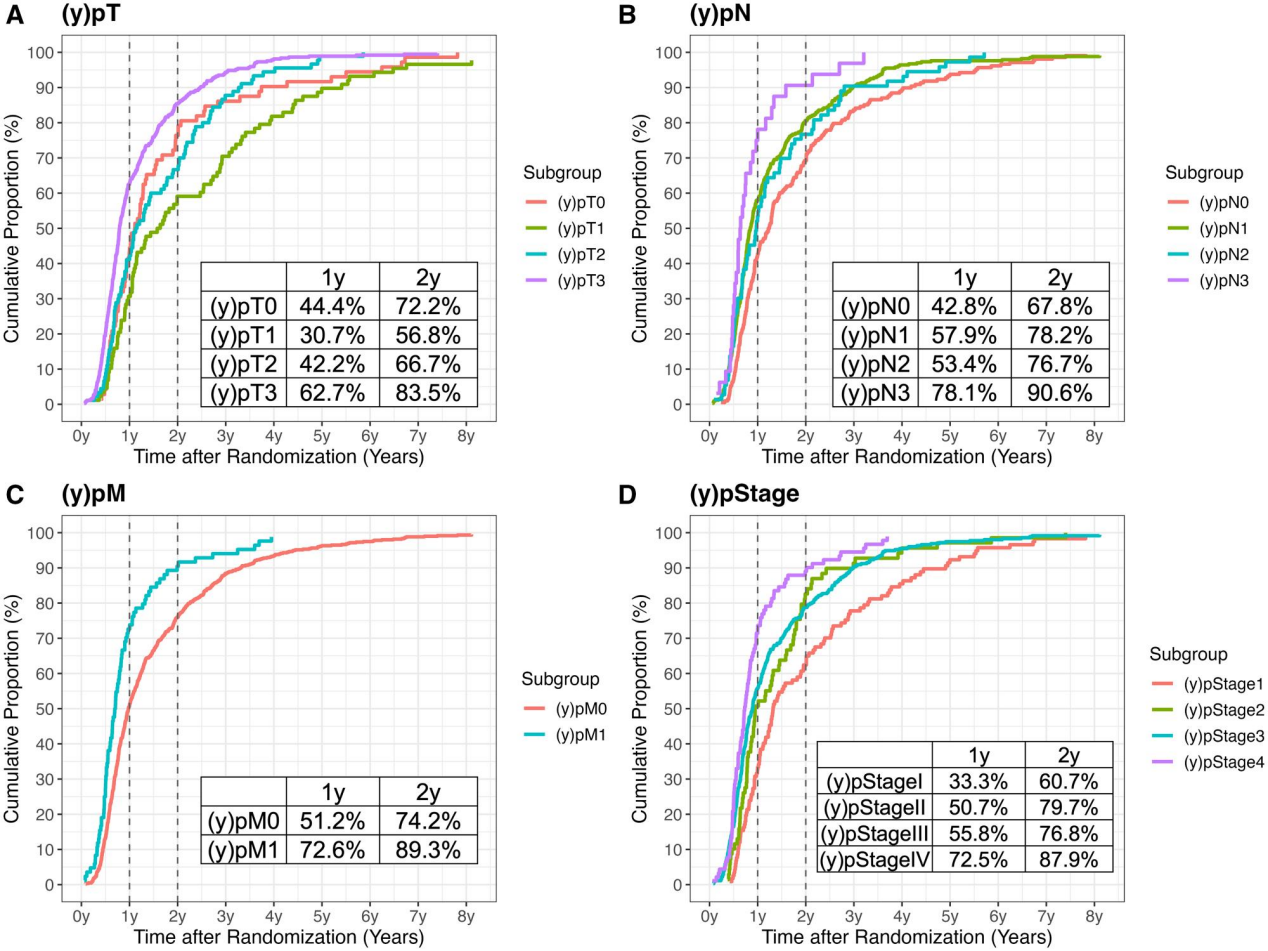
Cumulative incidence of recurrence by pathological subgroup in patients with squamous cell carcinoma.

Among patients with SCC who experienced recurrence, those with pN3 disease showed earlier recurrence patterns, with 78.1% recurring within 1 year and 90.6% within 2 years of randomization, compared with 42.8% and 67.8% in the pN0 group. Similar trends were observed in other subgroups, with patients who had more advanced-stage disease tending to experience earlier recurrence when recurrence occurred (Figure 3). Results for patients with adenocarcinoma are shown in Figure S3, demonstrating the same pattern.

**Figure 4.**
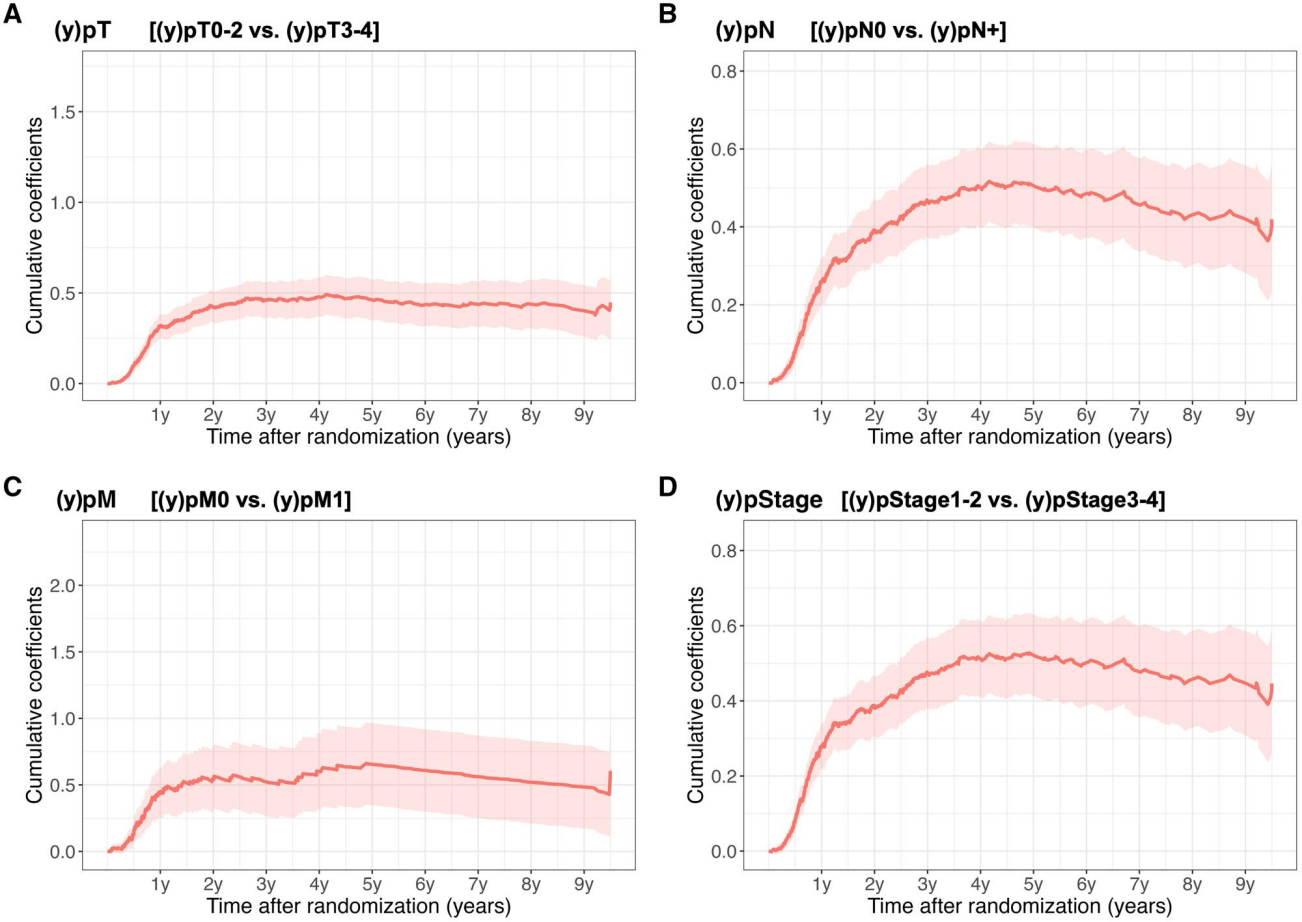
Time-dependent effects of pathological factors on recurrence-free survival estimated by Aalen’s additive hazards model in patients with squamous cell carcinoma. Note: To depict changes in hazard ratios with (y)pTNM as covariates, all variables under consideration were converted into binary variables. The less advanced category was used as the reference. Positive cumulative slopes indicate an increasing hazard over time, whereas flat or decreasing slopes suggest the attenuation of prognostic impact. This time-varying analysis illustrates how the influence of each pathological factor on recurrence risk dynamically changes postoperatively.

In the analysis using Aalen’s additive model (Figure 4), among patients with SCC, the cumulative coefficients for (y)pT, (y)pN, (y)pM, and (y)pStage all remained positive over time, indicating that more advanced categories were associated with an increased excess hazard of recurrence. Although the effect sizes increased during the first few years after randomization, they generally plateaued thereafter. Notably, for pN and pStage, the slope turned negative approximately 4 years after randomization. This finding suggests that, compared with patients with pN0 disease, the excess hazard associated with pN-positive disease diminished after a certain period after randomization. In contrast, in the analysis of adenocarcinoma (Figure S4), the slope did not shift from positive to negative; however, similar to SCC, a plateau was observed approximately 4 years after randomization.

## Discussion

In other malignancies such as colorectal cancer, melanoma, and lung cancer, the hazard of recurrence or death has been shown to be relatively high during the first few years after diagnosis and to decline markedly thereafter.^12^^,^^13^^,^^23^^,^^38^^,^^39^ In the present CS analysis, a similar trend was observed in esophageal cancer, and this finding was consistently supported by the results from Aalen’s additive hazards model. Traditionally, survival in many cancers has been reported from the time of diagnosis, such as 5-year OS or 5-year RFS. However, such estimates may not accurately represent the prognosis for patients who have already survived for a certain period after their initial diagnosis and treatment. For these patients, CS provides a more appropriate reflection of how time-dependent changes in hazard influence prognosis. In other words, CS analysis offers more meaningful prognostic information by realistically estimating the likelihood of future survival for patients who have successfully passed through the initial phase of cancer treatment. This study represents the first large-scale report using IPD from RCTs to examine the patterns and timing of recurrence and survival in esophageal cancer, including patients from both Eastern and Western populations, thereby providing important insights into its prognostic dynamics.

According to the results of a nationwide survey conducted in Japan on follow-up practices after curative treatment for esophageal cancer, among patients with pathological stage II-IV disease, 64% of institutions conducted 5 or more outpatient visits in the first year, 61% conducted 3 or more visits in the fifth year, and 76% conducted at least 1 visit in the 10th year. Regarding imaging, 61% of institutions performed 3 or more computed tomography scans from the neck to the pelvis in the first year, whereas at least 1 scan was performed by 96% and 59% of institutions in the fifth and 10th years, respectively.^40^ Additionally, studies have compared the prognosis between clinically indicated imaging and routine surveillance imaging,^41^ whereas other studies have reported the outcomes of intensive follow-up at 6-month intervals.^42^ However, follow-up strategies after curative resection for esophageal cancer continue to be determined by each institution, and, to our knowledge, no reports have clarified the utility of regular follow-up or identified the most effective surveillance methods.^43^ Given this situation, the findings from the present study—that conditional RFS improves substantially over time, particularly in patients with advanced-stage disease—suggest that prognostic predictions or follow-up planning should not rely solely on baseline stage. Simultaneously, these results may serve as a useful basis for tailoring survivorship care in patients who, despite being diagnosed with advanced disease, continue to remain recurrence-free for several years.

In previous CS analyses, preoperative and postoperative stages were commonly used as covariates. The novelty of the present study lies in its exploratory analysis of CS differences according to response to preoperative treatment, including pCR, pGrade, and TRG (Figure 2 and Figure S2). In the comparison between pCR and non-pCR, similar to (y)pTNM stage, we found in both SCC and adenocarcinoma that, although patients without pCR initially had poorer outcomes, their prognosis tended to become comparable to, or even surpass, that of patients with pCR once they reached RFS_5_|RFS_4_. These results provide encouraging insights: although patients with a favorable response to preoperative therapy clearly demonstrate better prognosis when measured from the time of surgery, those with a poor response may experience narrowing of the prognostic gap if they remain recurrence-free for several postoperative years. Furthermore, previous research has shown that combining pStage and pGrade offers superior prognostic discrimination than pStage alone.^44^ This suggests that postoperative information reflecting treatment response may also serve as a useful marker for optimizing follow-up strategies after curative resection.

This study had several limitations. First, in the CS analysis, the number of patients at risk in each group decreased over time owing to recurrence, death, or censoring. Although the present analysis encompassed up to RFS_5_|RFS_4_, the patients included in this assessment were required to have at least 9 years of follow-up after randomization, a criterion met by 10.3% of all patients with SCC (Figure 1). Consequently, the confidence intervals inevitably widened at later time points, necessitating caution is warranted when interpreting the results from the later follow-up periods. Nevertheless, a strength of this study is that it was conducted as a secondary analysis of RCTs, where the frequency of right-censoring is relatively limited. Second, information on recurrence patterns was not always sufficiently available. As shown in Table S2, the proportion of distant metastases among recurrences was 60.5% in SCC and 78.1% in adenocarcinoma. However, because some trials did not provide data on recurrence patterns, these proportions were estimated only from trials with available information. Therefore, a detailed analysis by recurrence pattern could not be performed. Another limitation is the potential heterogeneity across the 11 trials included in this integrated analysis. Although treatment regimens somewhat varied among studies, approximately 75% of patients received neoadjuvant therapy (Table 1), which represents the standard of care for SCC and adenocarcinoma. Therefore, this variability could not have markedly influenced the overall findings. However, the limited number of patients who underwent surgery alone or received adjuvant chemotherapy precluded subgroup analyses in these specific populations.

Conditional RFS improved over time in patients with esophageal cancer, especially in those with advanced (y)pTNM stage. Although patients with more advanced disease are usually monitored more intensively after surgery, these findings suggest that similar follow-up intensity could be aligned across stages once patients remain recurrence-free for a certain postoperative period. Additionally, these findings may provide valuable insights for guiding survivorship care in patients who remain recurrence-free for several years, even if they were initially diagnosed with advanced disease.

## Supplementary Material

djaf347_Supplementary_Data

## Data Availability

This study conducted an integrated analysis by collecting individual patient data (IPD) from randomized controlled trials (RCTs). The principal institution, Keio University, obtained approval from its Institutional Review Board (IRB). Keio University established data-sharing agreements with each participating institution and received the data accordingly. Therefore, the data from this study cannot be shared with third parties, even upon request.
