# Supplementary material for: Conditional recurrence-free survival after curative esophagectomy: individual patient data analysis of 11 trials
Source: J Natl Cancer Inst. 2026 Jan 8;118(4):737–45. doi: 10.1093/jnci/djaf347 (PMC13064524; doi:10.1093/jnci/djaf347)
Supplement: djaf347_Supplementary_Data [file djaf347_supplementary_data.docx]

**Supplemental Appendix:**

Okui J, et al. Conditional recurrence-free survival after curative esophagectomy: Individual patient data analysis of 11 trials.

**Contents**

| **Items** | **Title** | **Page** |
| --- | --- | --- |
| **Figure S1** | Patient flow diagram | 2 |
| **Figure S2** | Conditional 5-year recurrence-free survival by landmark time and subgroup in patients with adenocarcinoma | 3 |
| **Figure S3** | Cumulative incidence of recurrence by pathological subgroup in patients with adenocarcinoma | 4 |
| **Figure S4** | Time-dependent effects of pathological factors on recurrence-free survival estimated by Aalen’s additive hazards model in patients with adenocarcinoma | 5 |
| **Table S1** | Eligible studies identified by systematic review | 6 |
| **Table S2** | Event incidence and recurrence pattern distribution by histologic type | 8 |
| **Table S3** | Median follow-up duration and interquartile range for each conditional RFS cohort | 9 |
| **Table S4** | Proportions of cancer- and non-cancer-related deaths over time | 10 |
| **Study Protocol** | | 11 |

# Figure S1. Patient flow diagram.

Abbreviations: EC, esophageal cancer; EGJ, esophagogastric junction; ICI, immune checkpoint inhibitor; IPD, individual patient data.


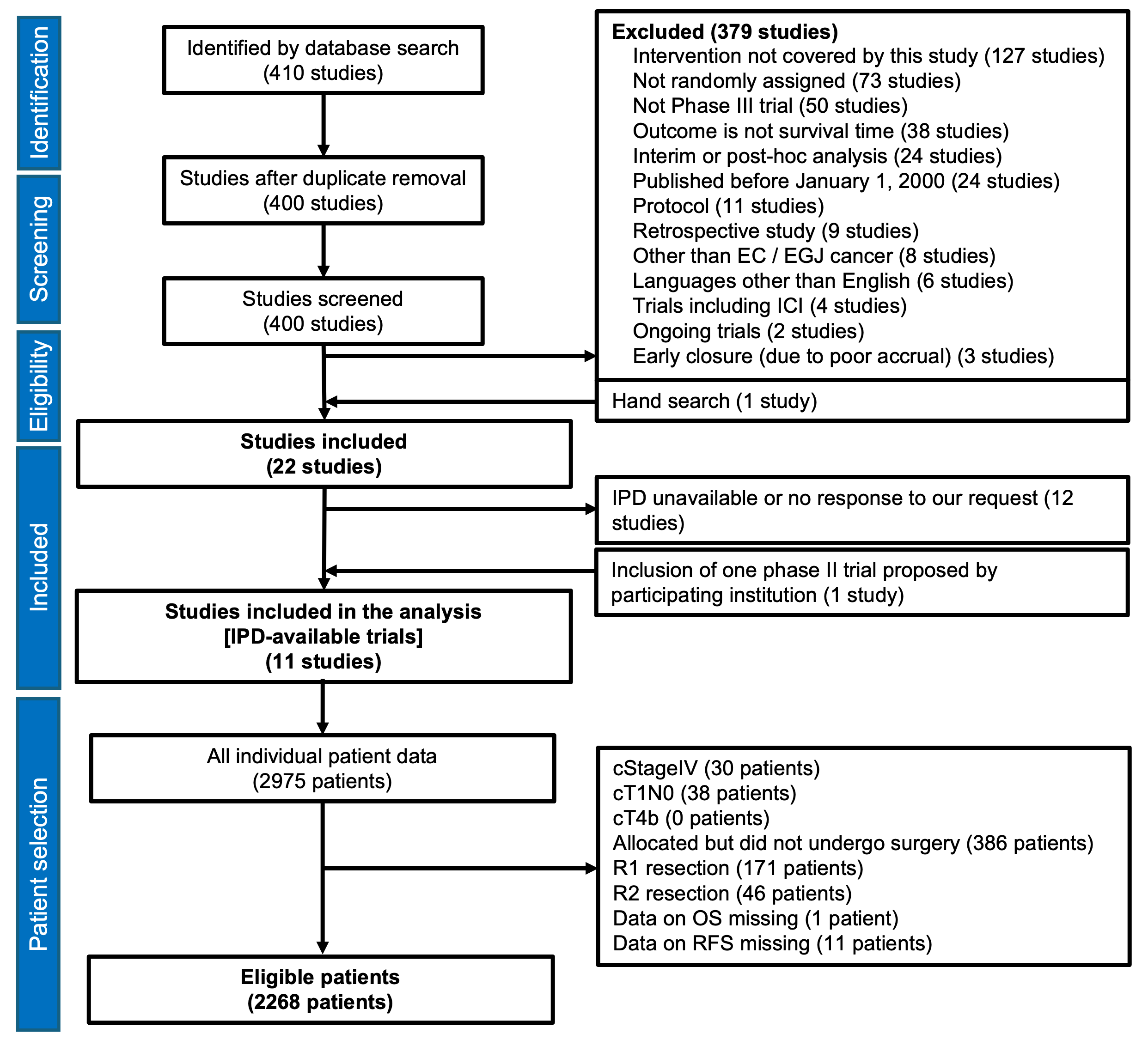


**Figure S2. Conditional 5-year recurrence-free survival by landmark time and subgroup in patients with adenocarcinoma.**


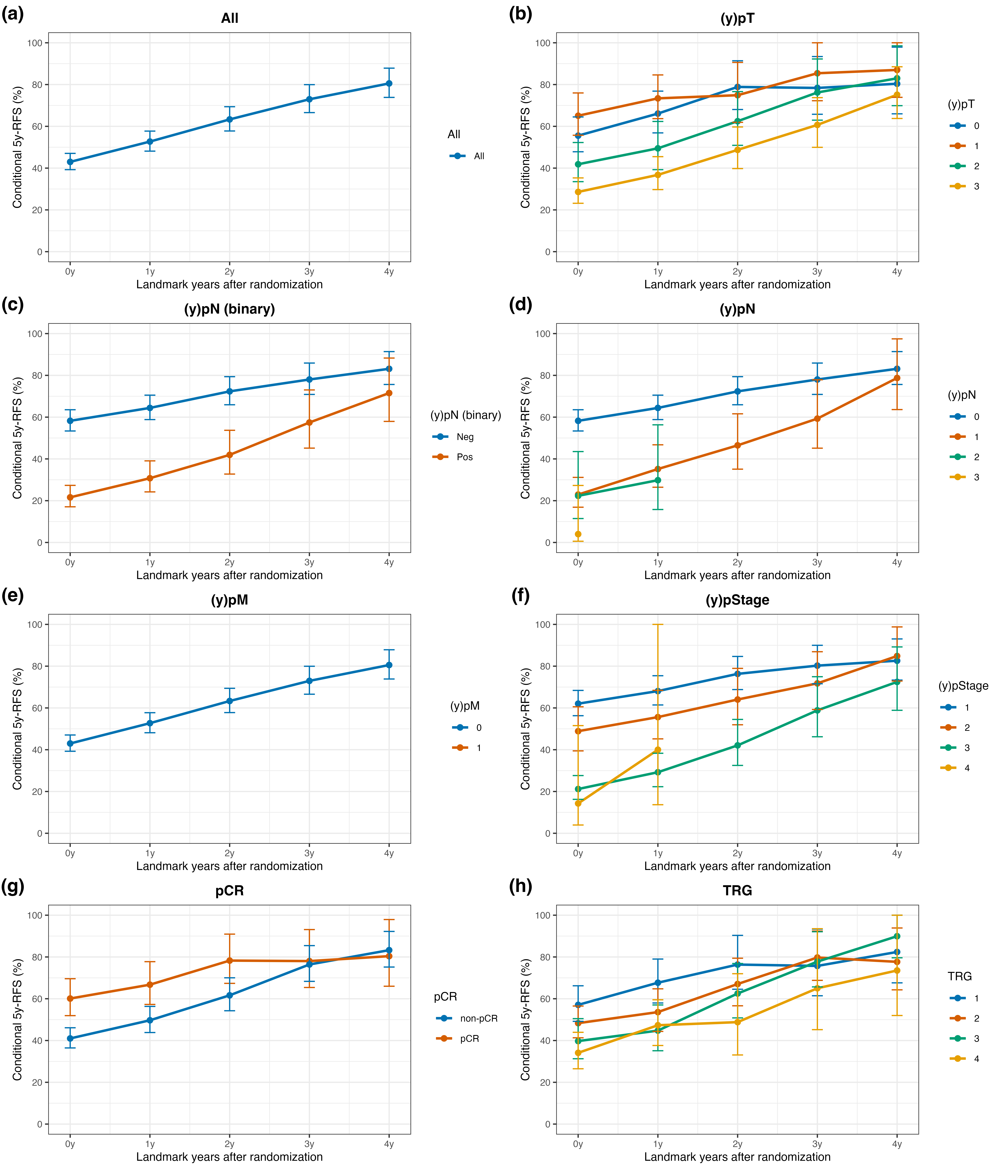


Abbreviations: pCR, pathological complete response; pGrade, pathological grade; RFS, recurrence-free survival; TRG, tumor regression grade.

Note: As no patients with adenocarcinoma had (y)pM disease, a comparison between (y)pM0 and (y)pM1 was not possible.

**Figure S3. Cumulative incidence of recurrence by pathological subgroup in patients with adenocarcinoma.**

Note: Since there were no patients with pM disease in the adenocarcinoma group, a comparison between pM0 and pM1 could not be performed.


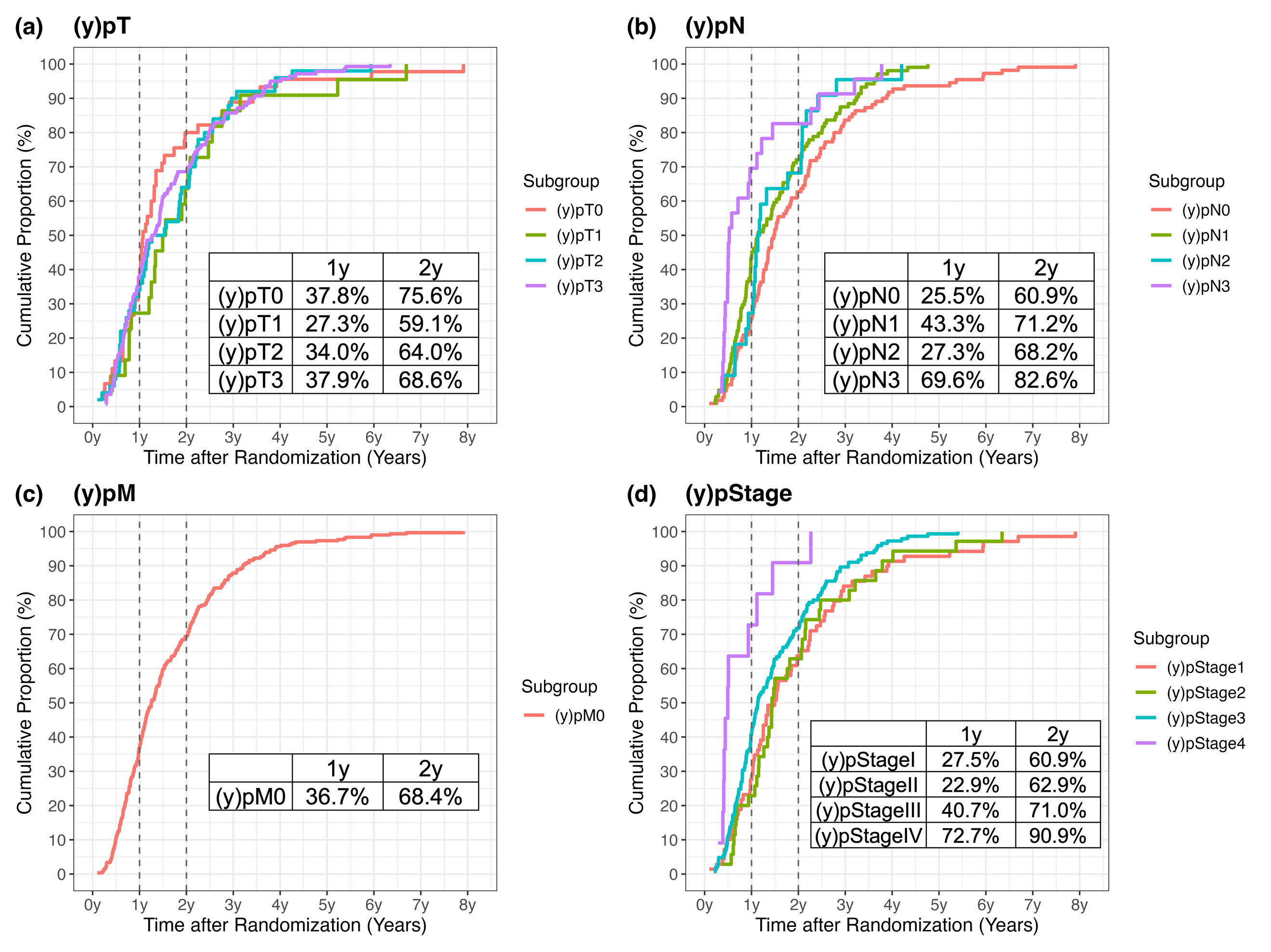


# Figure S4. Time-dependent effects of pathological factors on recurrence-free survival estimated by Aalen’s additive hazards model in patients with adenocarcinoma.

Note: Since there were no patients with pM disease in the adenocarcinoma group, a comparison between (y)pM0 and (y)pM1 could not be performed.

To depict changes in hazard ratios with (y)pTNM as covariates, all variables under consideration were converted into binary variables. The less advanced category was used as the reference. Positive cumulative slopes indicate an increasing hazard over time, whereas flat or decreasing slopes suggest attenuation of prognostic impact. This time-varying analysis illustrates how the influence of each pathological factor on recurrence risk dynamically changes throughout the postoperative course.


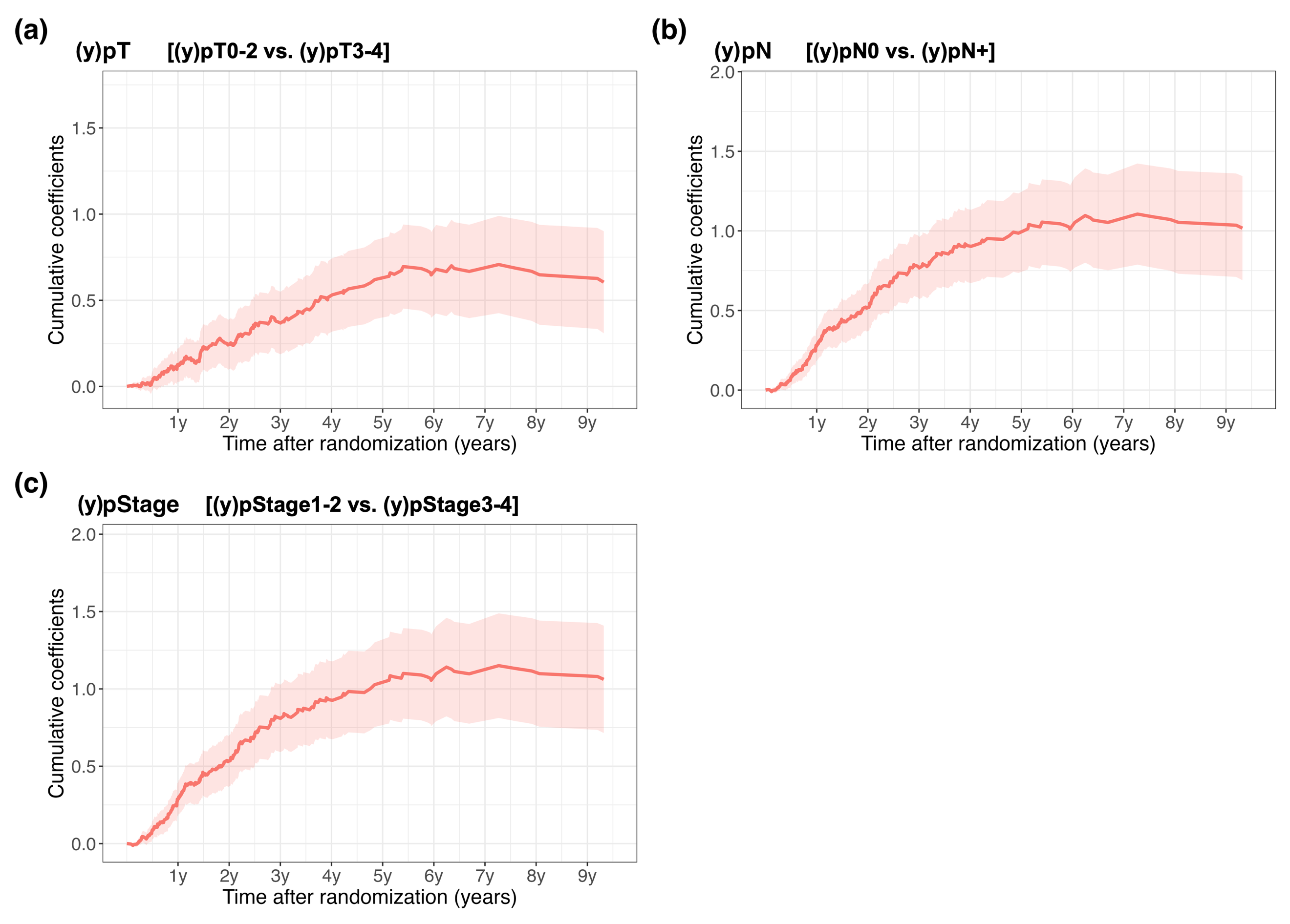


# Table S1. Eligible studies identified by systematic review.

| **Trial** | **Author** | **Published** | **Modality** | **RT** | **IPD** | **n** | **GEJC** | **SCC** | **AC** |
| --- | --- | --- | --- | --- | --- | --- | --- | --- | --- |
| JCOG1109* | Kato, et al. | 2024 | CS vs. CS | No | **Yes** | 401 | No | Yes | No |
| JCOG1109* | Kato, et al. | 2024 | CS vs. CRS | Yes | **Yes** | 399 | No | Yes | No |
| NeoRes2 | Nilsson, et al. | 2023 | CRS vs. CRS | Yes | **Yes** | 249 | Yes | Yes | Yes |
| Neo-AEGIS | ReyNolds, et al. | 2023 | CS vs. CRS | Yes | No | 362 | Yes | No | Yes |
| CMISG1701 | Tang, et al. | 2022 | CS vs. CRS | Yes | **Yes** | 264 | No | Yes | No |
| FLOT4 | Al-Batran, et al. | 2019 | CSC vs. CSC | No | No | 716 | Yes | No | Yes |
| SAKK75/08 | Ruhstaller, et al. | 2018 | CRS vs. CRS | Yes | **Yes** | 300 | Yes | Yes | Yes |
| NEOCRTEC5010 | Yang, et al. | 2018 | S vs. CRS | Yes | No | 451 | No | Yes | No |
| ST03 | Cunningham, et al. | 2017 | CSC vs. CSC | No | No | 1063 | Yes | No | Yes |
| OE05 | Alderson, et al. | 2017 | CS vs. CS | No | No | 897 | Yes | No | Yes |
| NeoRes** | Klevebro, et al. | 2016 | CS vs. CRS | Yes | **Yes** | 181 | Yes | Yes | Yes |
| Zhao, et al. | Zhao, et al. | 2015 | CS vs. CSC | No | No | 346 | No | Yes | No |
| FFCD9901 | Mariette, et al. | 2014 | S vs. CRS | Yes | **Yes** | 195 | No | Yes | Yes |
| JCOG9907 | Ando, et al. | 2012 | CS vs. SC | No | **Yes** | 330 | No | Yes | No |
| CROSS | van Hagen, et al. | 2012 | S vs. CRS | Yes | **Yes** | 366 | Yes | Yes | Yes |
| Boonstra, et al. | Boonstra, et al. | 2011 | S vs. CS | No | **Yes** | 169 | No | Yes | No |
| FFCD9703 | Ychou, et al. | 2011 | S vs. CS | No | No | 224 | Yes | No | Yes |
| OE02 | Allum, et al. | 2009 | S vs. CS | No | No | 802 | No | Yes | Yes |
| FFCD9102 | Bedenne, et al. | 2007 | CRS vs. CRS | Yes | **Yes** | 259 | No | Yes | Yes |
| MAGIC | Cunningham, et al. | 2006 | S vs. CSC | No | No | 503 | Yes | No | Yes |
| Burmeister, et al. | Burmeister, et al. | 2005 | S vs. CRS | Yes | No | 256 | No | Yes | Yes |
| Lee, et al. | Lee, et al. | 2004 | S vs. CS | No | No | 101 | No | Yes | No |
| JCOG9204 | Ando, et al. | 2003 | S vs. SC | No | **Yes** | 242 | No | Yes | No |
| MRC | MRC | 2002 | S vs. CS | No | No | 802 | No | Yes | Yes |

**Abbreviations**: AC, adenocarcinoma; RT, radiotherapy; SCC, squamous cell carcinoma.

**Abbreviations of “Modality”**: C, Chemotherapy; R, radiotherapy; S, surgery.

CS, Neoadjuvant chemotherapy followed by surgery; CRS, Neoadjuvant chemoradiotherapy followed by surgery; S, Surgery alone; SC, Surgery followed by adjuvant chemotherapy.

***Note**: JCOG1109 included three arms (one control arm and two test arms), and therefore it is presented in two rows in the table. ****Note**: Only the NeoRes trial is a phase II trial.

(Cont.)

| **Trial** | **Author** | **Published** | **HR_OS_** | **(95% CI)** | | **HR_RFS_** | **(95% CI)** | |
| --- | --- | --- | --- | --- | --- | --- | --- | --- |
| JCOG1109* | Kato, et al. | 2024 | 0.69 | 0.52 | 0.93 | 0.69 | 0.53 | 0.90 |
| JCOG1109* | Kato, et al. | 2024 | 0.87 | 0.66 | 1.15 | 0.80 | 0.62 | 1.04 |
| NeoRes2 | Nilsson, et al. | 2023 | 1.35 | 0.94 | 1.95 | 1.18 | 0.85 | 1.66 |
| Neo-AEGIS | ReyNolds, et al. | 2023 | 1.03 | 0.77 | 1.38 | 0.89 | 0.68 | 1.17 |
| CMISG1701 | Tang, et al. | 2022 | 0.82 | 0.58 | 1.18 | 1.07 | 0.71 | 1.60 |
| FLOT4 | Al-Batran, et al. | 2019 | 0.77 | 0.63 | 0.94 | 0.75 | 0.62 | 0.91 |
| SAKK75/08 | Ruhstaller, et al. | 2018 | 0.79 | 0.57 | 1.08 | 0.76 | 0.56 | 1.02 |
| NEOCRTEC5010 | Yang, et al. | 2018 | 0.71 | 0.53 | 0.96 | 0.58 | 0.43 | 0.78 |
| ST03 | Cunningham, et al. | 2017 | 1.08 | 0.91 | 1.29 | 1.04 | 0.89 | 1.22 |
| OE05 | Alderson, et al. | 2017 | 0.90 | 0.77 | 1.05 | 0.86 | 0.74 | 1.00 |
| NeoRes** | Klevebro, et al. | 2016 | 1.00 | 0.69 | 1.48 | 0.94 | 0.65 | 1.36 |
| Zhao, et al. | Zhao, et al. | 2015 | 0.79 | 0.59 | 0.95 | 0.62 | 0.49 | 0.73 |
| FFCD9901 | Mariette, et al. | 2014 | 0.96 | 0.69 | 1.34 | 0.95 | 0.68 | 1.31 |
| JCOG9907 | Ando, et al. | 2012 | 0.73 | 0.54 | 0.99 | 0.84 | 0.63 | 1.11 |
| CROSS | van Hagen, et al. | 2012 | 0.70 | 0.55 | 0.89 | 0.66 | 0.52 | 0.84 |
| Boonstra, et al. | Boonstra, et al. | 2011 | 0.68 | 0.48 | 0.96 | 0.66 | 0.47 | 0.93 |
| FFCD9703 | Ychou, et al. | 2011 | 0.69 | 0.5 | 0.95 | 0.65 | 0.48 | 0.89 |
| OE02 | Allum, et al. | 2009 | 0.84 | 0.72 | 0.98 | 0.82 | 0.71 | 0.95 |
| FFCD9102 | Bedenne, et al. | 2007 | 0.91 | 0.68 | 1.22 | 1.07 | 0.81 | 1.41 |
| MAGIC | Cunningham, et al. | 2006 | 0.75 | 0.6 | 0.93 | 0.66 | 0.53 | 0.81 |
| Burmeister, et al. | Burmeister, et al. | 2005 | 0.89 | 0.67 | 1.19 | 0.82 | 0.61 | 1.10 |
| Lee, et al. | Lee, et al. | 2004 | 0.88 | 0.48 | 1.63 | 0.98 | 0.55 | 1.72 |
| JCOG9204 | Ando, et al. | 2003 | 0.81 | 0.57 | 1.16 | 0.70 | 0.50 | 0.98 |
| MRC | MRC | 2002 | 0.79 | 0.67 | 0.93 | 0.75 | 0.63 | 0.89 |

**Abbreviations**: AC, adenocarcinoma; RT, radiotherapy; SCC, squamous cell carcinoma.

**Abbreviations of “Modality”**: C, Chemotherapy; R, radiotherapy; S, surgery.

CS, Neoadjuvant chemotherapy followed by surgery; CRS, Neoadjuvant chemoradiotherapy followed by surgery; S, Surgery alone; SC, Surgery followed by adjuvant chemotherapy.

***Note**: JCOG1109 included three arms (one control arm and two test arms), and therefore it is presented in two rows in the table. ****Note**: Only the NeoRes trial is a phase II trial.

# Table S2. Event incidence and recurrence pattern distribution by histologic type.

|  | **Overall**  **n = 2268** | **SCC**  **n = 1597** | **Adenocarcinoma**  **n = 664** |
| --- | --- | --- | --- |
| Recurrence event | 982 (43.3%) | 680 (42.6%) | 297 (44.9%) |
| Death event | 1158 (51.1%) | 799 (50.0%) | 353 (53.2%) |
| RFS event | 1294 (57.1%) | 894 (56.0%) | 393 (59.2%) |
| Recurrence pattern*  Distant/locoregional (%) | 366/151 (70.8%/29.2%) | 130/85 (60.5%/39.5%) | 232/65  (78.1%/21.9%) |

**Abbreviations**: RFS, recurrence-free survival; SCC, squamous cell carcinoma.

*Note: In the recurrence pattern row, the count and proportion are based on patients who experienced recurrence and had documented information on the recurrence pattern, set as 100%.

**Table S3. Median follow-up duration and interquartile range for each conditional RFS cohort**

| **Histology** | **Cohort** | **Median Follow-up duration (days)** | **Interquartile range (days)** |
| --- | --- | --- | --- |
| SCC | RFS_5_\|RFS_0_ | 1155.0 | 330.0 - 2390.4 |
|  | RFS_5_\|RFS_1_ | 1517.0 | 602.0 - 2391.8 |
|  | RFS_5_\|RFS_2_ | 1426.0 | 697.0 - 2253.5 |
|  | RFS_5_\|RFS_3_ | 1267.0 | 668.0 – 1985.0 |
|  | RFS_5_\|RFS_4_ | 1070.6 | 519.8 - 1749.5 |
| Adenocarcinoma | RFS_5_\|RFS_0_ | 1002.0 | 375.8 - 1924.8 |
|  | RFS_5_\|RFS_1_ | 1027.5 | 417.8 – 1697.0 |
|  | RFS_5_\|RFS_2_ | 1054.0 | 425.0 - 1548.5 |
|  | RFS_5_\|RFS_3_ | 865.0 | 459.0 – 1623.0 |
|  | RFS_5_\|RFS_4_ | 651.0 | 403.5 – 2312.0 |

Abbreviations: RFS, recurrence-free survival; SCC, squamous cell carcinoma.

**Table S4. Proportions of cancer-related and non–cancer-related deaths over time.**

| **Histology** | **Cohort** | **Death** | **Cancer death** | **Non-cancer death** | **Unknown cause of death** | **Cancer death (%)** | **Non-cancer death (%)** |
| --- | --- | --- | --- | --- | --- | --- | --- |
| SCC | RFS_5_\|RFS_0_ | 677 | 487 | 160 | 30 | 75.3% | 24.7% |
|  | RFS_5_\|RFS_1_ | 327 | 203 | 106 | 18 | 65.7% | 34.3% |
|  | RFS_5_\|RFS_2_ | 176 | 82 | 85 | 9 | 49.1% | 50.9% |
|  | RFS_5_\|RFS_3_ | 103 | 39 | 58 | 6 | 40.2% | 59.8% |
|  | RFS_5_\|RFS_4_ | 68 | 20 | 44 | 4 | 31.3% | 68.8% |
| Adenocarcinoma | RFS_5_\|RFS_0_ | 155 | 99 | 41 | 15 | 70.7% | 29.3% |
|  | RFS_5_\|RFS_1_ | 83 | 57 | 17 | 9 | 77.0% | 23.0% |
|  | RFS_5_\|RFS_2_ | 41 | 26 | 9 | 6 | 74.3% | 25.7% |
|  | RFS_5_\|RFS_3_ | 16 | 9 | 5 | 2 | 64.3% | 35.7% |
|  | RFS_5_\|RFS_4_ | 8 | 4 | 3 | 1 | 57.1% | 42.9% |

Abbreviations: RFS, recurrence-free survival; SCC, squamous cell carcinoma.

# Study Protocol

The protocol begins on the next page.

Protocol

**Individual patient data (IPD) meta-analysis on surrogacy of disease-free survival for overall survival and**

**IPD network meta-analysis on comparison of perioperative multidisciplinary treatments**

**in resectable esophageal cancer and gastroesophageal junction cancer trials**

**JP-ESOP23 study**

Registered in PROSPERO: CRD42023396321

○ Coordinating investigator

Yuko Kitagawa

Department of Surgery, Keio University School of Medicine

○ Study executive committee

Satoru Matsuda

Department of Surgery, Keio University School of Medicine, Japan

Jun Okui

Department of Surgery, Keio University School of Medicine, Japan

Department of Preventive Medicine and Public Health, Keio University School of Medicine, Japan

Kengo Nagashima

Biostatistics Unit, Clinical and Translational Research Center, Keio University Hospital, Japan

Ver 1.6 （October 3, 2023）

### 0. Brief Summary

**0.1. Aim**

This study aims to evaluate disease-free survival (DFS), recurrence-free survival (RFS), progression-free survival (PFS), and pathological complete response (pCR) as a surrogate endpoint for overall survival (OS) using individual patient data (IPD) from resectable thoracic esophageal cancer (EC) and gastroesophageal junction cancer trials assessing therapies in (neo)adjuvant and perioperative settings, separately for chemotherapy and chemoradiotherapy. Moreover, this study aims to compare the clinical outcomes of perioperative multidisciplinary treatments using IPD network meta-analysis.

**0.2. Significance**

A disadvantage of OS, which is considered the gold standard, is that it requires an extended follow-up period. We aim to contribute to shortening the development period by demonstrating surrogacy by DFS, RFS, PFS, and pCR using the latest trial’s IPDs including JCOG1109. To date, there have been no IPD-level studies focused on EC demonstrating surrogacy nor studies conducting network meta-analyses comparing various (neo)adjuvant/perioperative multidisciplinary treatments. This study will provide crucial evidence for clinical practice through robust statistical analysis.

**0.3. Study design**

Individual patient data meta-analysis

**0.4. Inclusion and exclusion criteria**

**Study Selection for systematic review**

All randomized phase III trials comparing therapies in (neo)adjuvant and perioperative settings for resectable thoracic esophageal cancer and esophagogastric junction cancer will be sought electronically from MEDLINE and the Cochrane Central Register of Controlled Trials. Trials were eligible if they were published before December 31, 2022, and were excluded when involving comparisons of immune checkpoint inhibitors.

**Inclusion criteria for IPD**

1. Aged over 18 years old
2. Patients with thoracic esophageal cancer or esophagogastric junction cancer
3. Histologically proven adenocarcinoma, squamous cell carcinoma, adenosquamous carcinoma, or basaloid cell carcinoma
4. Clinical stage I, II, III (excluding cT1N0 and cT4b), or IV due to supraclavicular LN metastasis based on the 8th UICC-TNM classification before treatment

**Exclusion criteria for IPD**

1. Subjects refusing to participate in this study (opting out)

**0.5. Expected number of patients enrolled and study period**

- Expected number of patients enrolled:

At least **3584 patients** (from **JCOG9204, JCOG9907, JCOG1109, NeoRes, NeoRes II, CROSS, KOK, CMISG1701, FFCD9901, FFCD9102, SAKK75/08 and NEOCRTEC5010** trials)

- Study period:

Date of approval by the Institutional review board of Keio University School of Medicine (June 1st, 2023) ~ March 31th, 2026

**0.6. Data collection**

For all individual patients included in all trials, we request the following data:

Hospital, randomization date, treatment allocated by randomization, date of last follow-up or death, survival status, cause of death, relapse status, and type and date of relapse if any.

**0.7. Statistical methods**

**Surrogacy analysis**

- For the individual level, Kendall’ Tau will be estimated using copula models[^1–3^](https://paperpile.com/c/9U1P7r/se1U+ubaF+qRU0) or a IPCW estimator[^4^](https://paperpile.com/c/9U1P7r/aVQh) to assess surrogacy between DFS, RFS, PFS and OS
- For the individual level, Kendall's Tau will be estimated using a modified IPCW estimator^4^ to uncensored binary variables to assess surrogacy between pCR and OS
- For the trial level, the coefficient of determination between the natural logarithm of the hazard ratios will be used to assess surrogacy between DFS, RFS, PFS and OS

**IPD network meta-analysis**

- IPD network meta-analysis will be performed to compare the clinical outcomes of (neo)adjuvant/perioperative multidisciplinary treatments

**Prognostic factor analysis**

- Univariate and multivariable Cox proportional hazard model for predicting DFS, RFS, PFS, pCR and OS will be developed

**0.8. Contact for queries**

35 Shinanomachi, Shinjuku-ku, Tokyo, 160-8582 35, Japan

Department of Surgery, Keio University School of Medicine

Satoru Matsuda

TEL : +81-3-5363-3802

FAX : +81-3-3355-4707

Email: [s.matsuda.a8@keio.jp](mailto:s.matsuda.a8@keio.jp)

Table of contents

[0. Brief Summary 2](#_Toc126932242)

[1. Summary 5](#_Toc126932243)

[2. Background and rationale for the study 6](#_Toc126932244)

[2.1. Background and clinical significance 6](#_Toc126932245)

[2.2. Rationale and significance of this study 11](#_Toc126932246)

[2.3. Risk and benefits to participants 12](#_Toc126932247)

[2.4. Ethical guidelines 12](#_Toc126932248)

[3. Aim 12](#_Toc126932249)

[4. Methods for Systematic Review 13](#_Toc126932250)

[4.1. Data Sources 13](#_Toc126932251)

[4.2. Eligibility Criteria 13](#_Toc126932252)

[4.3. Search Strategies 13](#_Toc126932253)

[4.4. Study Selection 14](#_Toc126932254)

[4.5. Data Extraction 14](#_Toc126932255)

[4.6. Variables 14](#_Toc126932256)

[4.7. Assessment of Study Quality and bias 14](#_Toc126932257)

[4.8. Registration 15](#_Toc126932258)

[5. Study design 15](#_Toc126932259)

[5.1. Endpoints 15](#_Toc126932260)

[5.2. Methodology 15](#_Toc126932261)

[5.3. Individual patient data 17](#_Toc126932262)

[5.4. Expected number of patients enrolled and study period 19](#_Toc126932263)

[5.5. Termination of study 19](#_Toc126932264)

[6. Inclusion and exclusion criteria for IPD 19](#_Toc126932265)

[7. Access and review of raw data 20](#_Toc126932266)

[8. Ethics 20](#_Toc126932267)

[9. Data treatment and preservation of records 22](#_Toc126932268)

[10. Economic burdens, insurance, and other measures for subjects 22](#_Toc126932269)

[11. Agreement over publication of study results 22](#_Toc126932270)

[12. References 22](#_Toc126932271)

### Revision History

| Version | Date | Changes |
| --- | --- | --- |
| 1.0 | 2023/02/18 | First edition created |
| 1.1 | 2023/04/12 | Addition of joint research institutions/researchersChanged inclusion/exclusion criteria and search strategies for systematic review |
| 1.2 | 2023/04/24 | Addition of joint research institutions/researchersThe title of the research series was named “JP-ESOP23” |
| 1.3 | 2023/06/05 | Addition of joint research institutions/researchers |
| 1.4 | 2023/07/23 | Addition of PROSPERO registration numberAddition of joint research institutions/researchersUpdated “5.3.2 Data collection” |
| 1.5 | 2023/08/30 | Addition of joint research institutions/researchersChanges to research timeline |
| 1.6 | 2023/10/06 | Addition of joint research institutions/researchersEdited "5.3.2. Data collection" |

### 1. Summary

**1.1. Title**

Individual patient data (IPD) meta-analysis on surrogacy of disease-free survival for overall survival and IPD network meta-analysis on comparison of perioperative multidisciplinary treatments in resectable esophageal cancer and gastroesophageal junction cancer trials

**1.2. Coordinating investigator**

Yuko Kitagawa

Department of Surgery, Keio University School of Medicine

TEL: +81-3-5363-3802

FAX: +81-3-3355-4707

**1.3. Participating institutions and Collaborating Principal Investigator**

**Institutions** **Collaborating Principal Investigators (Academic titles)**

Keio university Yuko Kitagawa (Professor)

National Cancer Center Hospital Ken Kato (Chief)

Karolinska Instituet Magnus Nilsson (Professor)

Erasmus University Medical Center Bas Wijnhoven (Professor)

Zhongshan Hospital of Fudan University Lijie Tan (Professor)

Fédération Francophone de Cancérologie Digestive　 Thomas Aparicio (Président du Bureau)

Swiss Group for Clinical Cancer Research Thomas Ruhstaller (Professor)

Sun Yat-sen University Cancer Center Hong Yang (Professor)

**1.4. Study executive office**

Satoru Matsuda

Department of Surgery, Keio University School of Medicine, Japan

Jun Okui

Department of Surgery, Keio University School of Medicine, Japan

Department of Preventive Medicine and Public Health, Keio University School of Medicine, Japan

Kengo Nagashima

Biostatistics Unit, Clinical and Translational Research Center, Keio University Hospital, Japan

**1.5. Collaborative investigator**

Ken Kato

Department of Head, Neck and Esophageal Medical Oncology, National Cancer Center Hospital, Japan

Junki Mizusawa

Biostatistics Section, National Cancer Center Hospital, Japan

Yasunori Sato

Department of Preventive Medicine and Public Health, Keio University School of Medicine, Japan

Magnus Nilsson

Fredrik Klevebro

Division of Surgery and Oncology, Karolinska Institutet, Sweden

Bas P.L. Wijnhoven

Bianca Mostert

Charlène van der Zijden

Erasmus University Medical Center, Netherlands

Lijie Tan

Jun Yin

Department of Thoracic Surgery, Zhongshan Hospital of Fudan University, China

Thomas Aparicio

Antoine Drouillard

Guillaume Piessen

Fédération Francophone de Cancérologie Digestive, France

Thomas Ruhstaller

Peter Thuss-Patience

Swiss Group for Clinical Cancer Research, Switzerland

Hong Yang

Sun Yat-sen University Cancer Center, China

### 2. Background and rationale for the study

### 2.1. Background and clinical significance

**2.1.1. Epidemiology**

Esophageal cancer (EC) ranks seventh in incidence and sixth in mortality among all cancers in the world[^5^](https://paperpile.com/c/9U1P7r/BeOu). In 2017, there were 11,568 deaths from esophageal cancer, accounting for 3.1% of all cancer deaths (data on deaths by vital statistics), and it is the seventh most common cause of cancer death in Japanese males. In the same year, the age-adjusted mortality rate (per 100,000 population) was found to be 7.4 in males and 1.2 in females, and in recent years, morbidity and mortality rates have trended horizontally. The peak age of esophageal cancer is in the seventies, rendering this disease relatively common among the elderly.

**2.1.2. Criteria and definitions used in this study**

The anatomical subsite and staging were evaluated according to the eighth edition of UICC-TNM classification for clinical classification, and the histological subtype was defined by the 12th edition of the Japanese Classification of Esophageal Cancer.

**2.1.3. Location of occupation**

**The 8th edition of UICC-TNM Classification**

Cervical esophagus (Ce)*: From the inferior border of cricoid cartilage to the thoracic entrance (upper border of manubrium: approximately 18 cm from incisors)

Thoracic esophagus (Te): Upper thoracic esophagus (Ut): from the thoracic inlet to the level of the carina (approximately 24 cm from the incisors)

Mid-thoracic esophagus (Mt): The upper half of the esophagus divided twice from the level of the carina to the esophagogastric junction (the lower edge is about 32 cm from the incisor)

Lower thoracic esophagus (Lt): approximately 8 cm in length, including the abdominal esophagus. 1/2 of the lower half of the esophagus from the level of the tracheal bifurcation to the esophagogastric junction (lower edge about 40 cm from the incisors)

*:In this study, cervical esophageal cancer is not included.

**Siewert classification of esophageal adenocarcinoma****

Type I: adenocarcinoma of the distal esophagus (epicenter of lesion 1-5 cm above GEJ)

Type II: adenocarcinoma of the cardia (epicenter of lesion up to 1 cm above and 2 cm below GEJ)

Type III: sub-cardial type adenocarcinoma (epicenter of lesion 2-5 cm below GEJ)

**: In Japan, Nishi's classification was employed by the Japanese Classification of Esophageal Cancer and Gastric Cancer to define five types of GEJ cancer characterized by diameters of 40 mm or less and an epicenter within 2 cm proximal or distal from the GEJ, irrespective of histological type. “E,” “EG,” “E = G,” “GE” and “G” are used to describe the subtype according to the epicenter location at the rostral and caudal portions of the GEJ. This study will include trials and patients who meet either definition of GEJ.

**2.1.4. Clinical classification**

**The 8th edition of UICC-TNM Classification**

T – Primary Tumor

TX: Primary tumor cannot be assessed

T0: No evidence of primary Tumor

Tis: Carcinoma in situ/high-grade dysplasia

T1: Tumor invades lamina propria, muscularis mucosae, or submucosa

T1a: Tumor invades lamina propria or muscularis mucosae

T 1 b: Tumor invades subserosa

T2: Tumor invades muscularis propria

T3: Tumor invades adventitia

T4: Tumor invades surrounding tissue

T4a: Tumor invades the pleura, pericardium, and phrenic

T4b: Tumor invades other surrounding tissues, including the aorta, vertebral body, and trachea

N - Regional Lymph Nodes

NX: Regional lymph node metastasis cannot be assessed

N0: no regional lymph node metastases

N1: Metastasis in 1-2 regional lymph nodes

N2: Metastasis in 3-6 regional lymph nodes

N3: Metastases in 7 or more regional lymph nodes

M - Distant Metastasis

M0: no distant metastasis

M1: Distant metastasis

M1 may be identified using the following symbols:

Lung, PUL; bone marrow, MAR; bone, OSS; pleural, PLE; liver, HEP; peritoneum, PER; brain, BRA; adrenal gland, ADR

Lymph nodes, LYM; Skin, SKI; Other, OTH

**2.1.5. Definition of regional lymph nodes**

Irrespective of their primary site, regional lymph nodes are the nodes corresponding to the lymphatic flow region of the esophagus, including celiac axis and cervical paraesophageal lymph nodes, but not the supraclavicular lymph nodes.

In this study, regional lymph nodes (N1) of the 8th edition of TNM-UICC were defined using the lymph node names of the 12th edition of Japanese classification of esophageal cancer as follows: The following definitions and lymph node names are used for clinical staging, tumor response assessment, and radiation therapy field setting.

Table 2.1.5 Correspondence between regional lymph nodes in the thoracic esophagus (the 8th edition of UICC-TNM Classification) and lymph nodes in the esophageal cancer handling convention

| Lymph node name: (The 12th edition of Japanese classification of esophageal cancer)  () Inside the node code | Regional Lymph Nodes in the Thoracic Esophagus (Upper, Middle, and Lower)  (The 8th edition of TNM-UICC classification) |
| --- | --- |
| **Neck** | |
| ・Cervical paraesophageal lymph node (101). | Cervical paraoesophageal lymph nodes |
| ・Deep cervical lymph node (102-up, 102-mid) |  |
| ・Lymph nodes (103) around the pharynx |  |
| ・Supraclavicular lymph node (104) |  |
| **Chest** |  |
| ・Upper thoracic paraesophageal lymph node (105). | Upper thoracic paraesophageal lymph node (above the azygos vein) |
| ・Thoracic tracheal lymph nodes (106-rec, 106-pre, 106-tb). | Mediastinal lymph node |
| ・Bifurcational lymph nodes (107) | Bifurcational lymph nodes |
| ・Mid-thoracic paraesophageal lymph node (108) | Lower thoracic paraesophageal lymph node (below the azygos vein) |
| ・Main subbronchial lymph node (109) | Bifurcational lymph nodes |
| ・Lower thoracic paraesophageal lymph node (110) | Lower thoracic paraesophageal lymph node (below the azygos vein) |
| ・Supraphrenic lymph node (111) | Mediastinal lymph node |
| ・Posterior mediastinal lymph node (112-ao、112-pul) |  |
| ・Lymph node of ligamentum arteriosum (113) |  |
| ・Anterior mediastinal lymph node (114) |  |
| **Abdomen** | |
| ・Right cardia lymph node (1) | Perigastric lymph nodes excluding celiac artery lymph nodes |
| ・Left cardiac lymph nodes (2) |  |
| ・Lymph node of lesser curvature (3) |  |
| ・Greater curvature lymph nodes (4) |  |
| ・Suprapyloric lymph node (5) |  |
| ・Nodi subpylorici (6) |  |
| ・Left gastric trunk lymph node (7) | Perigastric lymph nodes excluding celiac artery lymph nodes |
| ・Common hepatic artery trunk lymph node (8) |  |
| ・Celiac periarterial lymph nodes (9) | Lymph nodes of celiac trunk |
| ・Subdiaphragmatic node (19) | Perigastric lymph nodes excluding celiac artery lymph nodes |
| ・Lymph nodes in the esophageal hiatus (20) | Mediastinal lymph node |

Shaded areas: According to the TNM classification, nodes do not fall into regional lymph nodes (N1) of the thoracic esophagus. Metastasis in these nodes is considered distant metastasis (M1).

All lymph node metastases other than those listed in Table 2.1.5 are considered distant metastases (M1).

**2.1.6. Clinical staging**

**The 8th edition of UICC-TNM Classification**

| Stage | T | N | M |
| --- | --- | --- | --- |
| 0 | Tis | N0 | M0 |
| I | T1 | N0 | M0 |
|  | T1 | N1 | M0 |
| II | T2 | N0, N1 | M0 |
|  | T3 | N0 | M0 |
| III | T1, T2 | N2 | M0 |
|  | T3 | N1, N2 | M0 |
| IVA | T4a, T4b | N0, N1, N2 | M0 |
|  | Regardless of T | N3 | M0 |
| IVB | Regardless of T | Regardless of N | M1 |

*cM1 due to #102 mid (mid-deep cervical lymph node) or #104（supraclavicular lymph node） metastasis were not included to this study.

**2.1.7. Pathological staging**

**The 8th edition of UICC-TNM Classification**

| Stage | T | N | M |
| --- | --- | --- | --- |
| 0 | Tis | N0 | M0 |
| IA | T1a | N0 | M0 |
| IB | T1b | N0 | M0 |
| IIA | T2 | N0 | M0 |
| IIB | T1 | N1 | M0 |
|  | T3 | N0 | M0 |
| IIIA | T1 | N2 | M0 |
|  | T2 | N1 | M0 |
| IIIB | T2 | N2 | M0 |
|  | T3 | N1, N2 | M0 |
|  | T4a | N0, N1 | M0 |
| IVA | T4a | N2 | M0 |
|  | T4b | Regardless of N | M0 |
|  | Regardless of T | N3 | M0 |
| IVB | Regardless of T | Regardless of N | M1 |

**2.1.8. Histopathological findings**

**The 12th edition of Japanese classification of esophageal cancer**

1）  Vascular invasion (Ly/V)

i） Lymphatic invasion (Ly)

Ly0: No lymphatic invasion

Ly1: Lymphatic invasion

Ly1a: When lymphatic invasion is mild. Invasion of one or two lymphatic vessels

Ly1b: When lymphatic invasion is moderate. Intermediate between Ly1a and Ly1c

Ly1c: When lymphatic invasion is severe

ii）   Venous invasion (V)

V0: There is no venous invasion.

V1: Venous invasion

V1a: When venous invasion is mild. Invasion of one or two veins

V1b: When venous invasion is moderate. Intermediate between V1a and V1c

V1c3 When venous invasion is severe

2）   Histopathological criteria for the therapeutic effects of radiation therapy and chemotherapy

| Grade0: Ineffective | No therapeutic effect on cancer tissues or cells |
| --- | --- |
| Grade1: Moderately effective | Cancer tissues and cancer cells, even if some degenerative findings are observed, contain cancer cells (including those whose plasma is eosinophilic and vacuolated and whose nuclei are swollen) that are judged to be able to proliferate, which account for more than one-third of the cancers in tissue sections |
| Grade1a: Very mildly effective | When more than two-thirds of the cancer cells are judged to be able to proliferate |
| Grade1b: Mild efficacy | When more than one-third and less than one-third of cancer cells are judged to be able to grow |
| Grade2: fairly effective | When less than one-third of the cancer cells are judged to be able to proliferate and because they are cancerous cells that are inclined to break down of the nucleus |
| Grade3: Complete response | In the absence of any cancer cells that are judged to be able to grow, all are occupied only by cancer cells that are inclined to collapse, or only traces of cancer are seen |

**2.1.9. Histological Classification**

**The 12th edition of Japanese classification of esophageal cancer**

The subjects of this study are shaded areas.

I. Epithelial carcinoma        Malignant epithelial tumor

1. Carcinoma squamous cell carcinoma

a. Well differentiated well differentiated type

b. Moderately differentiated moderately differentiated type

c. Poorly differentiated poorly differentiated type

2. Basaloid (squamous) carcinoma basaloid (-squamous) cell carcinoma

3. Carcinosarcoma carcinosarcoma

4. Adenocarcinoma adenocarcinoma

a. Well differentiated well differentiated type

b. Moderately differentiated moderately differentiated type

c. Poorly differentiated poorly differentiated type

5. Adenosquamous carcinoma adenosquamous carcinoma

6. Mucoepidermoid carcinoma mucoepidermoid carcinoma

7. Adenoid cystic carcinoma adenoid cystic carcinoma

8. Endocrine Neoplasia endocrine cell tumor

a)Carcinoid tumor carcinoid tumor

b)Endocrine carcinoma endocrine cell carcinoma

9. Anaplastic carcinoma undifferentiated carcinoma

10. Unclassified carcinoma others

II. Non-epithelial neoplasia non-epithelial tumor

1. Smooth Muscle Neoplasms smooth muscle tumor

2. Gastrointestinal stromal tumor (GIST)

3. Neurogenic Neoplasms neurogenic tumor

Schwannoma schawannoma; neurofibroma neurofibroma; granular cell tumor granular cell tumor

4. Other hemangioma hemangioma, lymphangioma lymphangioma, lipoma lipoma, etc.

III. Lymphoid Neoplasms lymphoid tumor

According to the WHO classification.

IV. OTHER MALIGNANT NEOPLASMS

1. Malignant melanoma malignant melanoma

2. Other

### 2.2. Rationale and significance of this study

Esophageal cancer (EC) ranks seventh in incidence and sixth in mortality among all cancers in the world[^5^](https://paperpile.com/c/9U1P7r/BeOu). As EC can spread from the neck to abdomen even in the early stage and lead systemic progression[^6,7^](https://paperpile.com/c/9U1P7r/1cwF+oZH7), multidisciplinary treatment, consisting of surgery, chemotherapy, and radiotherapy, is required[^8,9^](https://paperpile.com/c/9U1P7r/5enX+7WNM). The mainstay of treatment for resectable EC in western societies is preoperative chemoradiotherapy followed by transthoracic esophagectomy, although pre- or perioperative chemotherapy is also used[^10,11^](https://paperpile.com/c/9U1P7r/aeQc+JaZO). In Japan, neoadjuvant chemotherapy (NAC) followed by esophagectomy with radical lymph node (LN) dissection has been shown to be beneficial for esophageal squamous cell carcinoma (ESCC)[^12^](https://paperpile.com/c/9U1P7r/iMLJ). Despite these multidisciplinary treatments, the risk of recurrence remains high, and systemic adjuvant therapies, including immune checkpoint inhibitors, are being investigated to improve efficacy and safety outcomes[^13,14^](https://paperpile.com/c/9U1P7r/Iuhu+9k9W). Further clinical trials of more effective adjuvant treatments are warranted for patients with resectable EC.

Currently, the OS rate has been the most frequently used metric for judging the success of a given treatment in randomized controlled trials. A disadvantage of the OS, which is considered the gold standard, is that it requires an extended follow-up period and larger trial populations to detect statistically significant and clinically meaningful benefits. Another disadvantage of OS is that it is potentially diluted by non-malignant causes of death and advances in treatment of relapsed or advanced disease. A way to address these challenges is to explore statistically appropriate and clinically relevant surrogate endpoints.

Reasonable candidates for surrogate endpoints of OS, such as DFS, PFS, and RFS, have been investigated in various tumor areas, including colorectal, breast, and lung cancers[^15–17^](https://paperpile.com/c/9U1P7r/btCy+9ZSp+RWct). A meta-analysis by Oba et al., using IPD from 14 RCTs of gastric cancer in adjuvant therapy, concluded that DFS is an acceptable surrogate for OS[^18^](https://paperpile.com/c/9U1P7r/6ie6). However, to the best of our knowledge, few studies have specifically examined the surrogate endpoints of patients with EC. Kataoka et al., evaluated the correlation between treatment effects on PFS and OS from 10 trials’ aggregate data in EC, and PFS did not appear to be an appropriate surrogate endpoint for OS[^19^](https://paperpile.com/c/9U1P7r/oJ5f). Ajani et al., demonstrated in a literature-based study that the HR of DFS and PFS correlated with the HR of OS and reported that it could be a predictor in the (neo)adjuvant and perioperative setting[^20^](https://paperpile.com/c/9U1P7r/xYsl). Of note, these two studies were literature-based studies, and did not use IPD. It is important to conduct studies using IPD because trial-level correlations and individual-level correlations may not match[^21^](https://paperpile.com/c/9U1P7r/LusU). An IPD-based study targeting gastroesophageal adenocarcinoma was conducted[^22^](https://paperpile.com/c/9U1P7r/zIO7), however, the challenge is that there is no IPD study that validates the surrogate endpoint in EC. Additionally, Cools-Lartigue et al., reported in a cohort study that the prognosis of pCR differed depending on whether patients received NAC or NACRT as neoadjuvant therapy[^23^](https://paperpile.com/c/9U1P7r/6mLc). Therefore, when investigating pCR surrogacy, it is necessary to analyze each neoadjuvant modality separately.

The purpose of this study is to evaluate DFS, RFS, PFS, and pCR as a surrogate endpoint for OS using IPD from resectable EC trials assessing therapies in (neo)adjuvant and perioperative settings, separately for chemotherapy and chemoradiotherapy. We aim to contribute to shortening the development period by demonstrating surrogacy endpoints using the latest trial’s IPDs including JCOG1109.

### 2.3. Risk and benefits to participants

In the present study, individual subjects will enjoy no benefit because it does not provide treatment nor does it involve any financial benefits. The study is undertaken within the framework of routine clinical practice, and there is no economic burden to individuals participating in this study. The information suppliers are therefore anticipated to suffer no medical disadvantage arising from their cooperation with this study. Meanwhile, although the findings from this study will not readily provide the information suppliers with any useful data pertaining to clinical practice, we may expect that the study will allow the society in general (to which the information suppliers belong) to utilize the respective reports in the diagnosis and treatment of cancers in the future.

The study may invade the privacy of participants because it uses clinical information. In consideration of this risk, anonymity is maintained by deleting their name, date of birth, and clinical care ID from each specimen. An anonymous ID, which is different from a medical record number, will be assigned to each patient. The cross-reference table, indicating our hospital’s medical record number corresponding to each anonymous patient ID, is apprehended by the Personal Information Administrator. All analytical steps at the collaborative study centers are performed in an anonymous manner, preventing direct linkage of clinical and personal information. Consequently, the name, date of birth, and clinical care ID of all participants are deleted during submission from the collaborative study centers to our hospital.

### 2.4. Ethical guidelines

Protection of subjects’ human rights, medical research, and conducts: This study is performed in compliance with the Ethical Guidelines for Medical and Biological Research Involving Human Subjects (published on March 23, 2021, in Japan, Ministry of Education, Culture, Sports, Science and Technology and Ministry of Health, Labor and Welfare (partially amended on March 10, 2022)) and the Declaration of Helsinki (modified in 2013). Personal information is managed appropriately in compliance with the Guidelines on the Personal Information Protection Act (General Rules), paying due care for protection of personal information.

### 3. Aim

**3.1. Aim**

First, this study aims to evaluate disease-free survival (DFS), recurrence-free survival (RFS), progression-free survival (PFS), and pathological complete response (pCR) as a surrogate endpoint for overall survival (OS) using individual patient data (IPD) from resectable thoracic esophageal cancer (EC) and gastroesophageal junction cancer trials assessing therapies in (neo)adjuvant and perioperative settings, separately for chemotherapy and chemoradiotherapy. Second, this study aims to compare the clinical outcomes of perioperative multidisciplinary treatments using network meta-analysis. Finally, this study aims to perform prognostic factor analysis associated with OS.

**3.2. Significance**

A disadvantage of OS, which is considered the gold standard, is that it requires an extended follow-up period. We aim to contribute to shortening the development period by demonstrating surrogacy by DFS, RFS, PFS, and pCR using the latest trial’s IPDs including JCOG1109. To date, there have been no IPD-level studies focused only on EC demonstrating surrogacy nor studies conducting network meta-analyses comparing various (neo)adjuvant/perioperative multidisciplinary treatments. This study will provide crucial evidence for clinical practice through robust statistical analysis.

### 4. Methods for Systematic Review

### 4.1. Data Sources

All randomized trials comparing therapies in (neo)adjuvant and perioperative settings for resectable esophageal cancer and esophagogastric junction cancer will be sought electronically from MEDLINE and the Cochrane Central Register of Controlled Trials. No restrictions on language will be applied.

### 4.2. Eligibility Criteria

**Inclusion criteria**

1. Randomized trials comparing therapies in (neo)adjuvant and perioperative settings for resectable thoracic esophageal cancer and esophagogastric junction cancer
2. Closed to patient accrual before December 31, 2020

**Exclusion criteria**

1. Involving comparisons of immune checkpoint inhibitors
2. Not randomized
3. Retrospective study
4. Phase II or pilot/exploratory trial, or Phase III with early closure (due to poor accrual)
5. Published before January 1, 2000

### 4.3. Search Strategies

Specific search strategy proposal is as follows (MEDLINE as an example):

(("Esophagectomy"[MH] OR "Esophageal Neoplasms/Surgery"[MH]) AND

("Combined Modality Therapy"[MH] OR "Antineoplastic Combined Chemotherapy Protocols"[MH])) AND

("Randomized Controlled Trial" [PT])

### 4.4. Study Selection

In the stage of screening, the two reviewers will independently review abstracts retrieved by the search, and determine whether it is "Include", "Maybe", or "Exclude". Disagreements were verified by the two reviewers, and all judgments were reconciled. If both reviewers agree that the trial does not meet eligibility criteria, the trial will be excluded. In the stage of full text review, the two reviewers will obtain the full text of all remaining articles and use the same eligibility criteria to determine which trials to exclude. Any disagreement will be resolved through discussion within the two reviewers, and all judgments were reconciled. The reviewers will also record the reasons for ineligibility for excluded studies.

### 4.5. Data Extraction

In the stage of full text review, we will design and use a structured data extraction form to ensure consistency of information. Information extracted includes study characteristics (i.e., the first author, publication year, journal), participant characteristics, intervention details and outcome measures. Two reviewers will ascertain that the data are entered correctly into the final data set. Finally, for each principal investigator of the included trials, we will request IPD sharing after the study protocol has been approved by the Institutional Review Board of Keio University School of Medicine.

### 4.6. Variables

See “5.3.2. Data collection”.

### 4.7. Assessment of Study Quality and bias

Two reviewers will independently assess the risk of bias in the included studies using the tool described in the Cochrane Collaboration Handbook (Higgins et al., 2011). If details regarding allocation concealment or other study characteristics are insufficient, we will contact principal investigators to obtain further information. Any disagreement for rating will be resolved through discussion within the two reviewers, and all ratings were reconciled.

### 4.8. Registration

After the study protocol review by the Institutional Review Board of Keio University School of Medicine, we will register the systematic review protocol with PROSPERO before starting the review (Registration number: CRD42023396321).

### 5. Study design

### 5.1. Endpoints

**Surrogacy analysis**

(1) Primary endpoint

Kendall’s Tau between DFS and OS

(2) Secondary endpoints

Kendall’s Tau between RFS, PFS and OS

Kendall's Tau's IPCW estimator between pCR and OS

**IPD network meta-analysis and Prognostic factor analysis**

(1) Primary endpoint: OS

(2) Secondary endpoints: DFS, RFS, PFS, and pCR

(3) Exploratory endpoints:

Incidence proportion of intraoperative/postoperative complications

Any hematological toxicities (≥ CTCAE v4.0 Grade3)

### 5.2. Methodology

**5.2.1. Statistical approach**

Patients’ background, clinicopathological factors, and treatment outcomes are investigated and sent to Keio University.

As the surrogacy analysis, Kendall’s Tau will be estimated using copula models[^1–3^](https://paperpile.com/c/9U1P7r/se1U+ubaF+qRU0) to assess surrogacy between DFS, RFS, PFS and OS for the individual level. Moreover, Kendall's Tau will be estimated using a modified IPCW estimator to uncensored binary variables to assess surrogacy between pCR and OS^4^. For the trial level, the coefficient of determination between the natural logarithm of the hazard ratios will be used to assess surrogacy between DFS, RFS, PFS and OS.

As an IPD network meta-analysis (NMA), two methods will be used: One-stage method and Two-stage method. As a One-stage IPD NMA performed as the primary analysis, IPD is treated as the “minimum unit of the analysis”. On the other hand, as a two-stage IPD NMA, IPD is summarized for each trial, and aggregated data are finally integrated. The latter method is used when trials for which an IPD was not provided are included. In the NMA, comparison of various (neo)adjuvant/perioperative multidisciplinary treatments will be performed. The following interventions will be included: neoadjuvant chemotherapy followed by surgery, neoadjuvant chemoradiotherapy followed by surgery, adjuvant chemotherapy after surgery, adjuvant chemoradiotherapy after surgery, perioperative chemotherapy, perioperative chemoradiotherapy, and surgery alone. Of particular interest in this study is the comparison of the therapeutic effects of neoadjuvant chemotherapy of DCF (docetaxel, cisplatin, and 5-FU) and neoadjuvant chemoradiotherapy (NACRT). Other comparisons include modality of (neo)adjuvant treatment (i.e., chemotherapy and chemoradiotherapy), timing of treatment (i.e., perioperative and (neo)adjuvant chemotherapy) and surgical approach (minimally invasive esophagectomy and thoracotomy). We will present the summary hazard ratios (for time-to-event variables) or odds ratios (for dichotomous variables) for all pairwise comparisons in a league table, and we will also estimate the prediction intervals. A ranking of the treatments using p-scores and the surface under the cumulative ranking curves will be evaluated under the consistency assumption. Additionally, if a sufficient sample size is obtained, treatment-by-covariate interactions will be investigated. To confirm the assumption that the results of the direct and indirect comparisons are consistent, the amount of inconsistency will be assessed globally and locally with the design-by-treatment interaction model and the loop-specific method, respectively. Moreover, if NMA fails to adequately estimate the comparison of each treatment effect, we perform a pairwise meta-analysis that utilizes multivariable models to account for trial-wise cluster effects. For each pair-wise comparison, we will synthesize data to obtain hazard ratios or odds ratios with its 95% credible intervals by using random effects model.

As the prognostic factor analysis, the individual patients’ clinicopathological factors, (neo)adjuvant regimens and surgical outcomes will be used as covariates. Univariate and multivariable Cox proportional hazard model for predicting DFS, RFS, PFS, pCR and OS will be developed.

**5.2.2. Heterogeneity (Assessment and Handling)**

In the NMA, the between-study variance and I-squared will be estimated to quantify overall heterogeneity and inconsistency. We expect small amounts of heterogeneity and inconsistency to be present given the variety of study settings we plan to include. We will explore whether treatment effects for the primary outcome are robust in subgroup analyses and network meta-regression using the following variables: (1) study year; (2) histological subtype; (3) tumor location, (4) surgical approach, etc., if necessary.

**5.2.3. Publication Bias Assessment**

We will use funnel plots^1-3)^ and Egger’s test to evaluate the impact of publication bias. Moreover, trim and fill method^4)^ will be used to trim outliers on funnel plots, to integrate effects, and to estimate center of effect. Trials will be imputed symmetrically for each estimated center of effect, and finally, a sensitivity analysis combining all trials will be performed to obtain the estimand that accounts for the impact of publication bias.

1. Peters JL, Sutton AJ, Jones DR, et al. Contour-enhanced meta-analysis funnel plots help distinguish publication bias from other causes of asymmetry. J Clin Epidemiol2008;61:991–6.
2. Chaimani A, Salanti G. Using network meta-analysis to evaluate the existence of small-study effects in a network of interventions. Research Synthesis Methods 2012; 3: 161–176.
3. Chaimani A, Higgins JPT, Mavridis D, Spyridonos P, Salanti G. Graphical tools for network meta-analysis in STATA. PloS One 2013; 8: e76654.
4. Duval, S. and Tweedie, R. (2000). A nonparametric “Trim and Fill” method of accounting for publication bias in meta-analysis. Journal of American Statistical Association 95, 89-98.

**5.2.4. Subgroups**

Patients included in this study are not a homogeneous group, therefore, subgroup analysis will be performed. Specifically, we will perform the following subgroup analysis:

- Histological subtype (e.g. squamous cell carcinoma and adenocarcinoma)
- Modality of (neo)adjuvant treatment (e.g. chemotherapy and chemoradiotherapy)
- Timing of treatment (e.g. perioperative and (neo)adjuvant chemotherapy)
- Tumor location (e.g. thoracic esophagus cancer and esophagogastric junction cancer)
- Surgical approach (e.g. minimally invasive esophagectomy and thoracotomy)

**5.2.5. Missing Data**

We will not impute missing outcomes in this study. Imputation of covariates will be performed as needed.

**5.2.6. Sensitivity Analysis**

We will perform subgroup analysis described in “5.2.4. Subgroups” section. In addition, sensitivity analysis limited to PPS patients will be performed as needed. A description of the sensitivity analysis for publication bias can be found in “5.2.3. Publication Bias Assessment”.

**5.2.7. Software**

All statistical analyses were performed using R statistical software v. 4.2.1 (R Foundation for Statistical Computing, Vienna, Austria). The R package “meta” and “netmeta” will be used to perform network meta-analysis.

1. Balduzzi S, Rücker G, Schwarzer G (2019), How to perform a meta-analysis with R: a practical tutorial, Evidence-Based Mental Health; 22: 153-160.
2. Schwarzer G. Network meta-analysis. In: Schwarzer G, Carpenter JR, Rücker G, eds. Meta-analysis with R. Berlin: Springer, 2015: 187-216.

### 5.3. Individual patient data

**5.3.1. Original data source**

Data files in which anonymized patients data are described are defined as the original data source.

**5.3.2. Data collection**

For all individual patients, the following variables will be extracted at each institution.

**Pretherapeutic patient background**

1. Sex
2. Age
3. Eastern Cooperative Oncology Group (ECOG) performance status (or any indicator of performance status; if available)
4. Body mass index (if available)
5. Smoking status (if available)
6. Laboratory findings (if available; pretherapeutic squamous cell carcinoma antigen level, hemoglobin level, and albumin level)
7. Main tumor location
8. clinical TNM, Stage

**Details of RCT**

1. Hospital (Anonymized institute ID [ex. Hospital 001, Hospital 002…etc.])
2. Treatment allocated by randomization

**Surgical outcomes**

1. Days from randomization to surgery (if available)
2. Intraoperative blood loss
3. Operative time
4. Types of surgery, reconstruction, resected organs
5. One- or two-step surgery
6. Approached (Open, VATS, Robotic assisted)
7. Details of lymph node dissection (Field of lymph node dissection, D1/D2/D3 or dissected lymph node stations)
8. Intraoperative complications (National Cancer Institute Common Terminology Criteria for Adverse Events version 4.0 [CTCAE v4.0]) * ≥Grade3 (yes/no; if available)

**Pathological outcomes**

1. Histology
2. Pathological TNM, Stage
3. Location of pathological lymph node metastasis
4. Number of lymph node metastasis for each station / Number of lymph nodes retrieved in total
5. Lymphovascular invasion
6. Infiltrative growth pattern (INF) (if available)
7. PM (proximal margin), DM (distal margin) (if available)
8. **Tumor regression grade (Mandard classification)**
9. R (Resection; R0/R1/R2)

**Complications (CTCAE v4.0)**

**If CTCAE grade is unavailable, only yes/no for complications is sufficient.**

1. Postoperative pneumonia ≥ Grade 3 (yes/no)
2. Postoperative anastomotic leakage ≥ Grade 3 (yes/no)
3. Postoperative recurrent laryngeal nerve palsy ≥ Grade 3 (yes/no)
4. Reoperation (yes/no)
5. Any postoperative death during regular hospital stay (yes/no)
6. Any hematological toxicities during (neo)adjuvant or perioperative treatment ≥ Grade 3 (yes/no)

**Long-term prognosis**

1. Days from randomization to last follow-up or death
2. Days from randomization to recurrence
3. Survival status
4. Cause of death
5. Relapse status
6. Type of relapse (Locoregional or Distant metastasis)

**5.3.3. Data control, preservation and disposal of records**

The data will be anonymized after registration at the Keio University. The cross-reference table, showing our hospital’s medical record number corresponding to each anonymous patient ID, is taken custody by the Personal Information Administrator.

With respect to the information obtained from collaborative study centers other than the Keio University, neither the patient name nor the medical record number will be registered in this study, and the cross-reference table for these patients will only be available to the study center supplying the information.

The information, etc. for the patients registered with this study need to be stored for a period of five years after completion of the clinical study. After the end of this period, it is recommended to store the information, etc. for as long as possible.

The data collected by the Study Secretariat are stored semi-permanently in view of the possible need for long-term follow-up, etc. The protocol and documents made public will be stored at the Study Secretariat as records of supplied information, etc.

Data control and preservation of records use lockable cabinets installed at the Office of Surgery Department (General/Gastrointestinal Surgery), Keio University School of Medicine (Controller: Satoru Matsuda, Department of Surgery (General/Gastrointestinal Surgery), Keio University School of Medicine).

The data and cross-reference table showing the patient information corresponding to the anonymous ID will be discarded after deletion of every identifiable personal information when necessary. Data in the form of paper will be discarded using a shredder.

**5.3.4. Data submission**

　Anonymized patients’ data will be sent to the Keio University from the collaborating institutions. Anonymous patient’s ID will be attached to the respective records.

### 5.4. Expected number of patients enrolled and study period

Expected number of patients enrolled:

At least **3584 patients** (from **JCOG9204, JCOG9907, JCOG1109, NeoRes, NeoRes II, CROSS, KOK, CMISG1701, FFCD9901, FFCD9102, SAKK75/08 and NEOCRTEC5010** trials)

May 2023: Approved by the IRB of the Keio University School of Medicine

June 2023 – December 2023: Data collection

January 2024 – April 2024: Data analysis

May 2024 – May 2025: Drafting of the manuscript and submission to scientific journal

### 5.5. Termination of study

This study will be terminated in the following occasions.

・Study aim is achieved earlier than the expected date of termination

・Principal investigator considers to terminate this study for any other reasons

### 6. Inclusion and exclusion criteria for IPD

**6.1. Inclusion criteria for IPD**

1. Aged over 18 years old
2. Patients with thoracic esophageal cancer or esophagogastric junction cancer
3. Histologically proven adenocarcinoma, squamous cell carcinoma, adenosquamous carcinoma, or basaloid cell carcinoma
4. Clinical stage I, II, III (excluding cT1N0 and cT4b), or IV due to supraclavicular LN metastasis based on the 8th UICC-TNM classification before treatment

**6.2. Exclusion criteria for IPD**

1. Subjects refusing to participate in this study (opting out)

### 7. Access and review of raw data

・Data will be handled at the Department of Surgery after anonymization.

・Access to the respective data will be restricted to the Study Representative, the Principal Investigators, the Sub-investigators, and the Personal Information Administrator.

・Raw data may be accessed during review by the Ethics Committee, etc. and inspection by the respective regulatory authorities.

### 8. Ethics

All researchers involved in this study are required to implement the study in compliance with the Declaration of Helsinki (October 2013, the version revised at Fortaleza) and the Ethical Guidelines for Medical and Biological Research Involving Human Subjects (made public on March 23, 2021, Ministry of Education, Culture, Sports, Science and Technology and Ministry of Health, Labor and Welfare (partially amended on March 10, 2022)).

**8.1. Informed consent**

Written informed consent will be waived because treatment will already have been completed for all subjects of the study, the study will only use anonymized patient information, and the study is designed as a retrospective observational study without any interventions or invasive procedures involved. After approval of this study by the Ethics Committee, a document will be made public on the homepage of the Keio University Hospital Clinical & Translational Research Center (https://www.ctr. hosp.keio.ac.jp/patients/optout/index.html) to provide information regarding the aims of this research and an opportunity of opting out.

Patients including in this study will have already received outpatient care at the collaborative study centers, and thus consent will be acquired during outpatient care unit visit. Regarding patients visiting the outpatient care unit at long intervals, having completed outpatient treatment, or died, the right to refuse participation in the study by means of opting out will be made public.

**8.2. Reporting to the head of each study center**

A report will be submitted in a prescribed format at 1-year (12 months) intervals from the date of study authorization.

**8.3. Disclosure of study-related information**

A document aimed at providing an opportunity of opting out will be made public on the homepage of the Keio University Hospital Clinical & Translational Research Center. The document will consist of the following information:

1. Outline and name of the study and approval of study implementation from the head of the study center.
2. The name of the study center and Principal Investigator (including the name of each collaborative study centers and the name of the Principal Investigator at each study center if the study is designed as a multicenter collaborative study).
3. Objectives and significance of the study
4. Study methods (including purposes and methods of using specimens/information collected from subjects) and study period.
5. Reasons for patient selection.
6. Burden on subjects and risks/benefits anticipated.
7. A statement that each subject can cancel their consent to participation or remain in the study any time after issuance.
8. A statement that refusal to participate or remain in the study or consent withdrawal will not cause any unfavorable treatment.
9. Methods for disclosing study-related information.
10. A statement that each subject can receive or access the documents related to the study protocol and study methods without compromising protection of personal information, etc. of other study subjects or compromising the novelty of the study, accompanied by information on how to receive or access such documents.
11. Treatment of personal information, etc. (including methods of anonymization, if necessary, and information on creating anonymous processed information or unidentifiable processed information, if necessary).
12. Methods for storage and disposal of specimens and information.
13. Sources of research funds and conflicts of interest related to research at the study centers involved as well as the status of personal benefits and conflicts of interest related to research conducted by the researchers involved.
14. How to treat the results, etc. of the study.
15. How to consult study subjects and individuals related to them (including genetic counseling).
16. A statement regarding potential economic burdens or awards, if any, to any study subjects.

**8.4.　Contact for queries**

35 Shinanomachi, Shinjuku-ku, Tokyo, 160-8582 35, Japan

Department of Surgery, Keio University School of Medicine

Satoru Matsuda

TEL: +81-3-5363-3802

FAX: +81-3-3355-4707

Email:　s.matsuda.a8@keio.jp

**8.5. Conflicts of interest**

Implementation of the present study does not involve any expenses. Should expenses for telecommunication, etc. arise, they will be paid from the “Budget for the Department of Surgery, Keio University School of Medicine.”

For researchers, conflicts of interest will be managed at each study center.

**8.6. Dealing with subjects after study completion**

After study completion, the Principal Investigator will be required to provide medical care to patients to whom he/she considers optimal while considering the outcomes of this study.

**8.7. Dealing with significant findings on genetic characteristics, etc.**

Not applicable to this study.

### 9. Data treatment and preservation of records

The data used will be stored at the Study Secretariat and may be used in future research studies. Future use of the obtained data will be limited to “Individual-patient-data (IPD) meta-analysis on Surrogacy of disease-free survival for overall survival and IPD network meta-analysis on Comparison of perioperative multidisciplinary treatments in resectable esophageal cancer and gastroesophageal junction cancer trials” and new research using such data will require approval by the Ethics Committee of the Keio University School of Medicine.

### 10. Economic burdens, insurance, and other measures for subjects

Subjects will be exempted from any economic burdens related to this study.

### 11. Agreement over publication of study results

The results of this study will be made public in the forms of presentations at professional society meetings and scientific papers. The methods of presentation, co-presenters, and co-authors will be organized by the coordinating investigator and study executive office following discussions among Principal Investigators.

### 12. References

1. Burzykowski T, Molenberghs G, Buyse M, et al. Validation of surrogate end points in multiple randomized clinical trials with failure time end points. *J R Stat Soc Ser C Appl Stat*. 2001;50:405–422.

2. Weber EM, Titman AC. Quantifying the association between progression-free survival and overall survival in oncology trials using Kendall’s τ. *Stat Med*. 2019;38:703–719.

3. Emura T, Sofeu CL, Rondeau V. Conditional copula models for correlated survival endpoints: Individual patient data meta-analysis of randomized controlled trials. *Stat Methods Med Res*. 2021;30:2634–2650.

4. Lakhal L, Rivest L-P, Beaudoin D. IPCW Estimator for Kendall’s Tau under Bivariate Censoring. *Int J Biostat*.;5 . Epub ahead of print February 4, 2009. DOI: 10.2202/1557-4679.1121.

5. Bray F, Ferlay J, Soerjomataram I, et al. Global cancer statistics 2018: GLOBOCAN estimates of incidence and mortality worldwide for 36 cancers in 185 countries. *CA Cancer J Clin*. 2018;68:394–424.

6. Akutsu Y, Kato K, Igaki H, et al. The Prevalence of Overall and Initial Lymph Node Metastases in Clinical T1N0 Thoracic Esophageal Cancer. *Ann Surg*. 2016;264:1009–1015.

7. Takeuchi H, Fujii H, Ando N, et al. Validation study of radio-guided sentinel lymph node navigation in esophageal cancer. *Ann Surg*. 2009;249:757–763.

8. Watanabe M, Otake R, Kozuki R, et al. Recent progress in multidisciplinary treatment for patients with esophageal cancer. *Surg Today*. 2020;50:12–20.

9. Matsuda S, Takeuchi H, Kawakubo H, et al. Current Advancement in Multidisciplinary Treatment for Resectable cStage II/III Esophageal Squamous Cell Carcinoma in Japan. *Ann Thorac Cardiovasc Surg*. 2016;22:275–283.

10. Lordick F, Mariette C, Haustermans K, et al. Oesophageal cancer: ESMO Clinical Practice Guidelines for diagnosis, treatment and follow-up†. *Ann Oncol*. 2016;27:v50–v57.

11. Ajani JA, D’Amico TA, Bentrem DJ, et al. Esophageal and Esophagogastric Junction Cancers, Version 2.2019, NCCN Clinical Practice Guidelines in Oncology. *J Natl Compr Canc Netw*. 2019;17:855–883.

12. Ando N, Kato H, Igaki H, et al. A Randomized Trial Comparing Postoperative Adjuvant Chemotherapy with Cisplatin and 5-Fluorouracil Versus Preoperative Chemotherapy for Localized Advanced Squamous Cell Carcinoma of the Thoracic Esophagus (JCOG9907). *Annals of Surgical Oncology*. 2012;19:68–74.

13. Kelly RJ, Ajani JA, Kuzdzal J, et al. Adjuvant Nivolumab in Resected Esophageal or Gastroesophageal Junction Cancer. *N Engl J Med*. 2021;384:1191–1203.

14. Doki Y, Ajani JA, Kato K, et al. Nivolumab Combination Therapy in Advanced Esophageal Squamous-Cell Carcinoma. *N Engl J Med*. 2022;386:449–462.

15. Buyse M, Burzykowski T, Michiels S, et al. Individual- and trial-level surrogacy in colorectal cancer. *Stat Methods Med Res*. 2008;17:467–475.

16. Burzykowski T, Buyse M, Piccart-Gebhart MJ, et al. Evaluation of tumor response, disease control, progression-free survival, and time to progression as potential surrogate end points in metastatic breast cancer. *J Clin Oncol*. 2008;26:1987–1992.

17. Mauguen A, Pignon J-P, Burdett S, et al. Surrogate endpoints for overall survival in chemotherapy and radiotherapy trials in operable and locally advanced lung cancer: a re-analysis of meta-analyses of individual patients’ data. *Lancet Oncol*. 2013;14:619–626.

18. Oba K, Paoletti X, Alberts S, et al. Disease-free survival as a surrogate for overall survival in adjuvant trials of gastric cancer: a meta-analysis. *J Natl Cancer Inst*. 2013;105:1600–1607.

19. Kataoka K, Nakamura K, Mizusawa J, et al. Surrogacy of progression-free survival (PFS) for overall survival (OS) in esophageal cancer trials with preoperative therapy: Literature-based meta-analysis. *Eur J Surg Oncol*. 2017;43:1956–1961.

20. Ajani JA, Leung L, Singh P, et al. Disease-free survival as a surrogate endpoint for overall survival in adults with resectable esophageal or gastroesophageal junction cancer: A correlation meta-analysis. *Eur J Cancer*. 2022;170:119–130.

21. Paoletti X, Lewsley L-A, Daniele G, et al. Assessment of Progression-Free Survival as a Surrogate End Point of Overall Survival in First-Line Treatment of Ovarian Cancer: A Systematic Review and Meta-analysis. *JAMA Netw Open*. 2020;3:e1918939.

22. Ronellenfitsch U, Jensen K, Seide S, et al. Disease-free survival as a surrogate for overall survival in neoadjuvant trials of gastroesophageal adenocarcinoma: Pooled analysis of individual patient data from randomised controlled trials. *Eur J Cancer*. 2019;123:101–111.

23. Cools-Lartigue J, Markar S, Mueller C, et al. An International Cohort Study of Prognosis Associated With Pathologically Complete Response Following Neoadjuvant Chemotherapy Versus Chemoradiotherapy of Surgical Treated Esophageal Adenocarcinoma. *Ann Surg*. 2022;276:799–805.
